# Visible Light‐Sensitive Sustainable Quantum Dot Crystals of Co/Mg Doped Natural Hydroxyapatite Possessing Antimicrobial Activity and Biocompatibility

**DOI:** 10.1002/smll.202405708

**Published:** 2024-10-24

**Authors:** Hossein Maleki‐Ghaleh, Bartosz Kamiński, Ehsan Moradpur‐Tari, Sada Raza, Mehdi Khanmohammadi, Rafał Zbonikowski, Mohammad Sadegh Shakeri, M. Hossein Siadati, Ali Akbari‐Fakhrabadi, Jan Paczesny

**Affiliations:** ^1^ Institute of Physical Chemistry Polish Academy of Sciences Kasprzaka 44/52 Warsaw 01–224 Poland; ^2^ Institute of Technology University of Tartu Nooruse 1 Tartu 50411 Estonia; ^3^ Biomaterials Group Materials Design Division Faculty of Materials Science and Engineering Warsaw University of Technology Wołoska 141 Warsaw 02–507 Poland; ^4^ Institute of Nuclear Physics Polish Academy of Sciences Krakow PL‐31342 Poland; ^5^ Materials Science and Engineering Faculty K. N. Toosi University of Technology Tehran 15418 Iran; ^6^ Advanced Materials Laboratory Department of Mechanical Engineering University of Chile Santiago 8370456 Chile

**Keywords:** antimicrobial, cobalt/magnesium co‐doped, hydroxyapatite, quantum dot crystals, solid‐state reaction

## Abstract

Cutting‐edge research in advanced materials is increasingly turning toward the development of novel multifunctional nanomaterials for use in high‐tech applications. This research uses the solid‐state method as a solvent‐free technique to create multifunctional quantum dot (QD) hydroxyapatite (HA) crystals from bovine bone waste. By incorporating cobalt (Co) and magnesium (Mg) into the HA structure, the crystallinity of the hexagonal HA nanoparticles (99.7%), showing QD crystals is enhanced. Oxygen vacancies on the surfaces of the HA nanoparticles contributed to their bandgap falling within the visible light range. In addition, the dopants substituted calcium in the HA crystal structure and generated a divalent oxidation state, shifting the bandgap of natural HA toward red wavelengths (3.26 to 1.94 eV). Moreover, the incorporation of Co led to magnetization within the HA structure through spin polarization. Additionally, the doped QD crystals of HA nanoparticles showed significant antimicrobial activity against *Escherichia coli*, *Staphylococcus aureus*, and bacteriophages MS2, particularly under visible light exposure. In short, the Co/Mg co‐doped HA nanoparticles exhibited ferromagnetic properties, sensitivity to visible light, biocompatibility, and considerable antimicrobial effects, establishing their potential as sustainable multifunctional materials for biomedical applications, especially in anti‐infection treatments.

## Graphical abstract

The synthesis of cobalt/magnesium‐doped hydroxyapatite, extracted from bio‐waste, using the solid‐state reaction method results in the production of nanoparticles composed of quantum dot crystals with a crystallinity of ≈99.7%. These nanoparticles possess ferromagnetic properties, visible light sensitivity, biocompatibility, and antibacterial effects, making them highly promising for advanced biomedical applications, particularly for anti‐infection purposes.

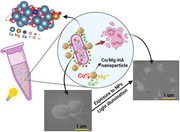

## 1 Introduction

Bacterial and viral infections are crucial challenges in the health and biomedical fields.^[^ ^1^, ^2^ ^]^ As an obvious example, transferring bacteria to the body environment during implant surgery can lead to infection, allergy, and tissue necrosis.^[^ ^2^, ^3^ ^]^ The decrease in the efficacy of antibiotics due to the bacteria's resistance has already increased the concern of dealing with microbes.^[^ ^4^ ^]^ Nanotechnology is one of the new fields in response to the challenging threat of resistance of pathogens to antibiotic drugs by identifying how the materials design can be used to synthesize nanoparticles for the therapeutic management of pathogen infections.^[^ ^5^, ^6^ ^]^ Using biocompatible novel nanostructured materials is a promising solution for dealing with bacterial infections.^[^ ^7^ ^]^ Inorganic biocompatible materials are particularly interesting in designing antibacterial nanoparticles due to their outstanding chemical stability and durability compared to organic materials.^[^ ^7^, ^8^ ^]^ In the meantime, hydroxyapatite (HA), an inorganic substance with biocompatibility and bioactivity qualities, is a suitable alternative for an antimicrobial base material.^[^ ^9^, ^10^, ^11^ ^]^ The electrostatic interaction between HA nanoparticles and the bacterial cell wall and the increase in oxidative stress are two crucial reasons for the antibacterial properties of HA nanoparticles.^[^ ^10^, ^11^ ^]^ More interestingly, the antimicrobial performance of HA can be intensified by altering its atomic structure with dopant elements.^[^ ^12^, ^13^, ^14^, ^15^, ^16^, ^17^ ^]^ Research studies regarding the effect of doping elements such as Zn,^[^ ^12^ ^]^ Cu,^[^ ^13^ ^]^ Mg,^[^ ^14^ ^]^ Co,^[^ ^15^ ^]^ Ge,^[^ ^16^ ^]^ and Ag^[^ ^17^ ^]^ have demonstrated that the antimicrobial behaviors of HA can be raised by more than 100%.

Modifying the atomic structure of HA with dopants can not only affect its biological performance but also change its physical properties, such as optical and magnetic properties.^[^ ^18^, ^19^ ^]^ Indeed, HA's optical and magnetic properties can be modified by purposeful design for the desired applications.^[^ ^18^, ^19^ ^]^ Studies to alter the optical properties of HA showed that doping elements, e.g., Se/Gd,^[^ ^20^ ^]^ Zn/Ni,^[^ ^21^ ^]^ and Sr/Mg,^[^ ^22^ ^]^ can significantly change the band gap of HA from 5.7 to 3.26 eV. Furthermore, some other dopants also provide magnetization; HA's magnetic saturation increased by more than 0.55 emu g^−1^ by doping it with Co and Fe.^[^ ^23^ ^]^ As a result of targeted doping of elements in the HA structure, not only can its antimicrobial features be significantly intensified, but also its optical and magnetic properties can be modified, transforming it from an insulator and non‐magnetic material into a semiconductor and ferromagnetic material, respectively.^[^ ^20^, ^21^, ^22^, ^23^ ^]^

HA is usually synthesized from chemical sources.^[^ ^24^, ^25^ ^]^ Producing HA from natural sources, such as extraction from the mammalian bones of cows, is easily possible.^[^ ^26^, ^27^ ^]^ Indeed, the extraction of HA from biowaste sources not only leads to energy savings due to needless preparation of chemical precursors but also because of the abundance of bone biowaste sources and its cheapness; it can be considered a sustainable and inexpensive way for producing HA.^[^ ^27^ ^]^ The HA extracted from natural sources can also be modified at the atomic level for the desired biological applications.^[^ ^28^ ^]^ For this purpose, the atomic structure of HA and, consequently, its physical and chemical characteristics can be altered using a solid‐state reaction method such as high‐energy ball milling (HEBM).^[^ ^28^, ^29^, ^30^ ^]^ Notably, particles obtained from the HEBM process can have a structure with nanometer crystals and high surface roughness.^[^ ^31^, ^32^, ^33^ ^]^ Crystalline HA interacts more with microorganisms and cell membranes than amorphous HA nanoparticles.^[^ ^34^, ^35^ ^]^ Likewise, by increasing the surface roughness, the interaction between nanoparticles and bacterial cell membranes intensifies, causing further enhancement in their overall antibacterial activities.^[^ ^36^, ^37^, ^38^, ^39^ ^]^

In the current study, for the first time, natural HA particles extracted from biowaste were doped with Co, Mg, and Co/Mg using HEBM. The structural characterization of the natural HA, Co‐HA, Mg‐HA, and Co/Mg‐HA nanoparticles was investigated using TEM, synchrotron SAXS‐WAXS, synchrotron XANES, and XPS analyses. Also, all four nanoparticles' optical and magnetic properties were analyzed using UV‐Vis DRS and VSM techniques. In addition, density functional theory (DFT) studies were performed on all four crystals to investigate the impact of Co and Mg dopants, particularly the O vacancy, on the structure of the natural HA. The antimicrobial activity of the nanoparticles was investigated against *Escherichia coli* (*E. coli*), *Staphylococcus aureus* (*S. aureus*), and bacteriophages MS2 under darkness and visible light irradiation conditions. Also, the biocompatibility of nanoparticles was studied using MTS assay, Alamar Blue assay, hemolysis, and Live/Dead assay.

## 2 Results and Discussion

### 2.1 Phase and Microstructural Evolution

**Figure 1:**
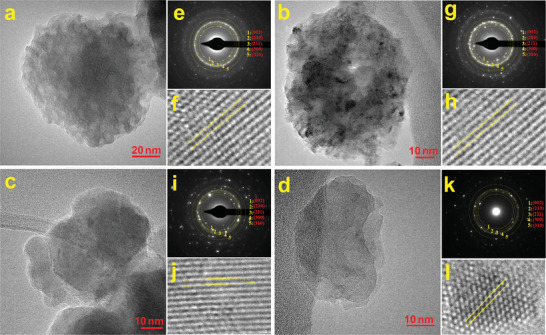
TEM images of the a) HA, b) Co‐H, c) Mg‐HA, and d) Co/Mg‐HA nanoparticles, SAED patterns of the e) HA, g) Co‐H, i) Mg‐HA, and k) Co/Mg‐HA nanoparticles, and HRTEM images of the f) HA, j) Co‐H, h) Mg‐HA, and l) Co/Mg‐HA nanoparticles.

**Figure** **1**a–d shows the transmission electron microscopy (TEM) images of HA (a), Co‐HA (b), Mg‐HA (c), and Co/Mg‐HA (d) nanoparticles prepared using HEBM, which have a flake‐like and quasi‐spherical shape. The selected area electron diffraction (SAED) patterns of the nanoparticles (Figure 1e,g,I,k) show the hexagonal crystal structure of HA^[^ ^40^ ^]^ with crystal planes (002), (210), (211), (300), and (130) for all samples so that the diffraction intensity increased due to Co, Mg, and Co/Mg dopants, indicating increase in crystallinity in the HA nanoparticles after doping with Co, Mg, and Co/Mg.

The bright‐field (BF) TEM images and high‐angle annular dark‐field scanning TEM (HAADF‐STEM) of the HA and Co/Mg‐HA nanoparticles are shown in Figure S1 (Supporting Information). The HRTEM images (Figure 1f,h,j,l) indicate that the distance between the atomic planes changed upon doping; distance between (002) atomic planes after Co, Mg, and Co/Mg doping decreased from 0.3435 to 0.3411, 0.3419, and 0.3413 nm, respectively. The lattice parameter c of HA decreased with Co and Mg doping, owing to the difference in ionic radius between Co^+2^ (70 pm), Mg^+2^ (72 pm), and Ca^+2^ (99 pm).^[^ ^41^, ^42^, ^43^ ^]^

Figure S2 (Supporting Information) displays scanning electron microscopy (SEM) images of the HA (a), Co‐HA (b), Mg‐HA (c), and Co/Mg‐HA (d). The nanoparticles exhibit a flake‐like, quasi‐spherical morphology consistent with TEM observations (Figure 1a–d). The size of all four nanoparticles was less than 80 nm, while the Co/Mg‐HA nanoparticles were smaller than other samples. Figure S3a (Supporting Information) presents the EDS elemental analysis of Co‐HA, Mg‐HA, and Co/Mg‐HA nanoparticles. The EDS spectra confirm the presence of Co, Mg, and Co/Mg elements in the Co‐HA, Mg‐HA, and Co/Mg‐HA nanoparticles, respectively, alongside Ca, P, and O elements. Besides, the EDS mapping, depicted in Figure S3b (Supporting Information), demonstrates a uniform distribution of Co, Mg, and Co/Mg elements within the respective nanoparticles following the HEBM process.

**Figure 2:**
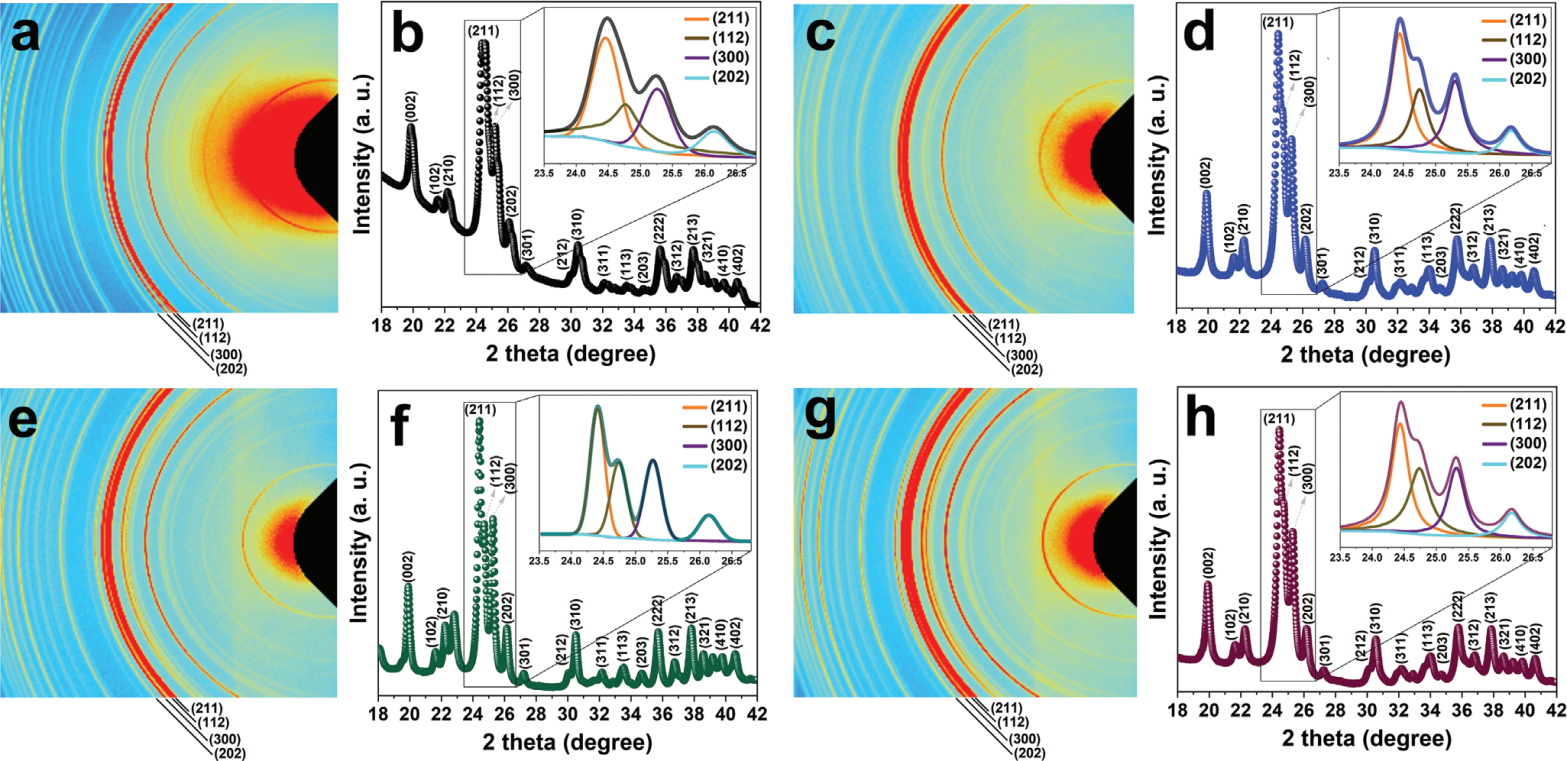
2D WAXS patterns of the a) HA, c) Co‐HA, e) Mg‐HA, and g) Co/Mg‐HA nanoparticles, 1D WAXS patterns of the b) HA, d) Co‐HA, f) Mg‐HA, and h) Co/Mg‐HA nanoparticles.

**Figure** **2** shows the 2D (a, c, e, g) and 1D (b, d, f, h) synchrotron wide‐angle X‐ray scattering (WAXS) patterns, revealing the hexagonal structure of all four nanoparticles according to JCPDS No. 9–432.^[^ ^44^ ^]^ The diffraction intensities of the crystal planes in 1D and 2D WAXS patterns were higher in the doped samples, indicating the rise in the crystallinity of the nanoparticles by the dopants. Regarding the quantitative indicator of HA crystallinity, the crystallinity index (CI) of HA nanoparticles was calculated using the CI = (I_300_ – V_112_/_300_)/I_300_ equation, where I300 is the intensity of the (300) reflection and V112/300 represents the intensity of the hollow between the (112) and (300) reflections in the 1D WAXS spectrum.^[^ ^45^ ^]^ The CI values for HA, Co‐HA, Mg‐HA, and Co/Mg‐HA were 88.9%, 93.3%, 98.1%, and 99.74%, respectively, indicating that the nanoparticle's crystallinity increased due to doping.

Although doping normally leads to defects and subsequent decrease in the crystallinity of the host material,^[^ ^46^ ^]^ in the present study, doping Co and Mg in the HA led to an increase in its crystallinity. This distinct behavior can be attributed to the solid‐state process and exothermic reactions between ceramic/metal particles during the HEBM process.^[^ ^47^ ^]^ As observed in the differential scanning calorimetry (DSC) curves shown in Figure S4 (Supporting Information), the two peaks at ≈615 and ≈650 °C in Mg‐HA, one peak at ≈835 °C in Co‐HA, and three peaks at ≈615, 650, and 835 °C in Co/Mg‐HA are related to exothermic reactions. Since in the HEBM process, the surface temperature of nanoparticles reaches more than 1000 °C, exothermic reactions within the Co/Mg and HA particles could lead to the recovery of the crystal defects and, subsequently, increase in the crystallinity of nanoparticles.

The 2D (a, c, e, g) and 1D (b, d, f, h) synchrotron small‐angle X‐ray scattering (SAXS) patterns for HA (a, b), Co‐HA (c, d), Mg‐HA (e, f), and Co/Mg‐HA (g, h) samples are displayed in Figure S5 (Supporting Information). The q values at the maximum intensity for all samples were in the range of 0.144 Å^−1^, indicating the porous structure of the nanoparticles.^[^ ^48^ ^]^ The natural bone structure contains numerous pores filled with organic materials such as cells and collagen.^[^ ^49^ ^]^ In the HEBM process, continuous crushing and sticking of particles led to a porous structure.^[^ ^50^ ^]^

The nanoparticles' radius of gyration (an indication of particle size) was determined by applying Guinier law to the 1D SAXS patterns displayed in Figure S6 (Supporting Information). The average size (R) of nanoparticles was calculated by the initial slope in the q^2^‐Ln I diagram and equation ln (I_q_) = e (q^2^) * Rg^2^/3 + ln (I_o_).^[^ ^48^ ^]^ From the initial slopes of the q^2^‐Ln I diagrams in Figure S6 (Supporting Information), R values of 77.5, 76.4, 64.3, and 62.6 nm for HA, Co‐HA, Mg‐HA, and Co/Mg‐HA samples, respectively, were calculated.

The geometric shape of the nanoparticles was investigated by applying the Porod law, using the Ln q‐Ln I diagrams derived from the 1D SAXS patterns (Figure S7, Supporting Information). The fractal dimension (α), represented as the initial slope of the scattering intensity curve (I(q) versus scattering vector q) using the power law,^[^ ^51^ ^]^ was considered to calculate the geometric shape of the particles. Accordingly, α values for HA, Co‐HA, Mg‐HA, and Co/Mg‐HA samples were 2.55, 2.61, 1.73, and 1.74, respectively. The α values 1, 2, and 3 correspond to spherical, disc, and rod shapes, respectively.^[^ ^51^ ^]^ Thus, HA and Co‐HA nanoparticles exhibited shapes between spherical and disc, and Mg‐HA and Co/Mg‐HA nanoparticles exhibited shapes between disc and rod.

TEM and SEM observations revealed that HA nanoparticles have a size range of 70–80 nm. The introduction of Co into the HA structure did not significantly alter the size or shape of the nanoparticles. However, doping HA with Mg and Mg/Co reduced particle size to 60–70 nm, and the morphology shifted from quasi‐spherical to rod‐like. Conventional techniques like SEM and TEM, though effective for assessing the morphology and size of nanoparticles, are inherently limited by the small sample sizes they analyze, leading to potential inaccuracies in determining the average particle size across large populations. In contrast, the synchrotron‐based SAXS technique offers a distinct advantage, allowing for the precise measurement of average nanoparticle sizes across billions of nanoparticles and providing a more accurate and statistically robust characterization.^[^ ^51^ ^]^

### 2.2 Evaluation of Chemical States and Electronic Properties

**Figure 3:**
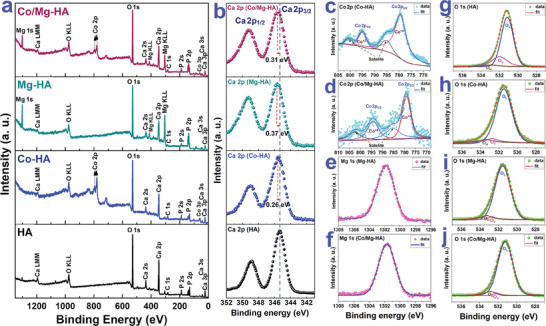
a) XPS survey spectra of the HA, Co‐HA, Mg‐HA, and Co/Mg‐HA nanoparticles, b) high‐resolution XPS spectra of the Ca 2p region for HA, Co‐HA, Mg‐HA, and Co/Mg‐HA nanoparticles, high‐resolution XPS spectra of the Co 2p region of the c) Co‐HA and d) Co/Mg‐HA nanoparticles, high‐resolution XPS spectra of the Mg 1s region for the e) Mg‐HA and f) Co/Mg‐HA nanoparticles, high‐resolution XPS of O1s of the g) HA, h) Co‐H, i) Mg‐HA, and j) Co/Mg‐HA nanoparticles.

The X‐ray photoelectron spectra (XPS) of the studied nanoparticles are displayed in **Figure** **3**a, manifesting the peaks associated with calcium (Ca), phosphorus (P), and oxygen (O). The Co and Mg elements were also present in the spectra of all doped samples. Figure 3b displays the high‐resolution XPS of the Ca 2p region for all four nanoparticles. For the HA nanoparticles, two significant peaks with binding energies of 347.2 and 350.8 eV, attributed to Ca 2p_3/2_ and Ca 2p_1/2_, respectively, related to the Ca bonds of HA,^[^ ^52^, ^53^ ^]^ were observed. Also, while the Ca 2p spectra for the doped samples were similar to the HA nanoparticles, there were slight differences in peak positions to higher binding energy states. These subtle differences could be due to the higher degree of crystallinity in the HA structure caused by doping.

**Figure 4:**
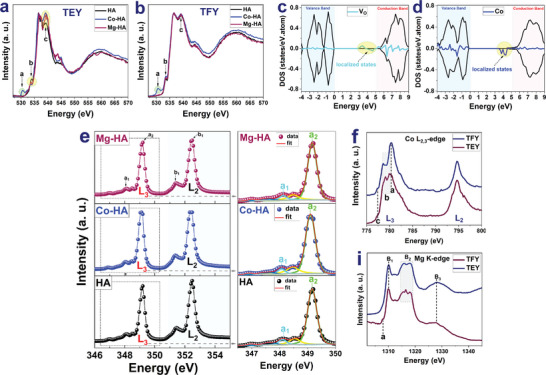
O K‐edge XANES of the HA, Co‐HA, and Mg‐HA nanoparticles in a) TEY and b) TFY modes, c) DOS diagram of the HA‐VO cell structure, d) DOS diagram of the Co‐doped HA cell structure, e) Calcium L‐edge TEY XANES spectra of HA, Co‐HA, and Mg‐HA nanoparticles, f) Co L‐edge XANES of the Co‐HA nanoparticles in TEY and TFY modes, g) Mg K‐edge XANES of the Mg‐HA nanoparticles in TEY and TFY modes.

Figure 4c,d depict the spectra of the Co 2p region for the Co‐HA and Co/Mg‐HA nanoparticles, respectively. In the high‐resolution Co 2p spectra (Figure 4c), Co 2p_1/2_ and Co 2p_3/2_ peaks were observed at 782.2 and 798.0 eV, respectively. The fitting peaks at 782.2 and 798.0 eV, along with their associated satellite peaks at 785.8 and 803.3 eV, pertained to Co^2+^, which conformed to the doping of Co^2+^ into the HA lattice.^[^ ^54^ ^]^

The spectra of the Mg 1s region for the Mg‐HA and Co/Mg‐HA nanoparticles are displayed in Figure 4e,f, respectively. The binding energy ≈1302.3 eV of Mg 1s spectra for both Mg‐HA and Co/Mg‐HA nanoparticles indicated Mg^2+^ oxidation states in the HA lattice, which correlated with Mg ions doped in the HA lattice.^[^ ^55^ ^]^

The spectra of the P 2p region are displayed in Figure S8 (Supporting Information). For the HA sample, binding energies of 132.9 and 134.1 eV belonging to P 2p_3/2_ and P 2p_1/2_, respectively, correspond to the phosphate group of HA.^[^ ^52^, ^53^ ^]^ Also, the P 2p spectra of the doped samples were similar to the P 2p spectra of HA nanoparticles, with the difference that the peak positions were slightly shifted to higher binding energy states, attributed to the higher crystallinity of the doped samples.

Figure 4g–j show the O 1s region for all four nanoparticles. The O 1s signal for all samples deconvoluted into three peaks: ≈531.1, 532.1, and 531.1 eV, which corresponded to oxygen lattice (O_L_), oxygen vacancies (O_V_), and hydroxyl groups (O_H_), respectively.^[^ ^56^ ^]^ HA nanoparticles had a higher concentration of oxygen vacancies than the other nanoparticles. Therefore, the concentration of O_V_ in HA decreased substantially upon doping Co, Mg, and Mg/Co.

The synchrotron X‐ray absorption near edge spectroscopy (XANES), utilizing fine structural characteristics at the absorption edge, reveals information such as electronic properties, geometry, and oxidation states. XANES is very sensitive to defects and atom symmetry in materials structure, and consequently, it has outstanding advantages in exploring structural defects in materials.^[^ ^57^ ^]^

**Figure** **4**a,b presents the O K‐edge XANES spectra of the HA, Co‐HA, and Mg‐HA nanoparticles in (a) TEY and (b) TFY modes, exhibiting main spectral peaks at 532.7, 535.0, 541.8, and 552.7 eV, characteristic of HA.^[^ ^58^ ^]^ The O K‐edge XANES spectra concern the transition of O 1s→2p, and the features near the edge originate from the hybridization of O 2p states with cationic states (p‐d hybridization), which diminishes the number of occupied O 2p orbitals and allows the dipole transition.^[^ ^58^ ^]^ As demonstrated in Figure 4a,b, the TEY and TFY spectra comprised the pre‐edge peak at ≈529.5 eV (feature b), arising from the excitation of the localized bound states caused by oxygen vacancies.^[^ ^59^ ^]^ Theoretically, oxygen vacancy leads to appearance of localized states between valance and conduction bands, which can manifest their presence in lower energy before prominent peaks in the spectrum of O K‐edge XANES as a pre‐edge peak.^[^ ^58^ ^]^ Figure 4c displays the density of states (DOS) diagram from DFT simulation results for the HA unit cell with oxygen vacancy. The states originated from oxygen vacancy (presented in the cyan spectrum) and the total DOS of HA atoms (in the black spectrum) for up and down spins. As shown in Figure 4c, the states arose due to oxygen vacancy below the conduction band (featured as localized states).

In TEY spectra (Figure 4a), doping Co and Mg into the HA nanoparticles diminished the intensity of feature b, demonstrating the reduction of notable oxygen vacancies.^[^ ^60^ ^]^ Also, one noticeable change in the TEY mode of O K‐edge was observed with the raised shoulder (feature c in Figure 4a) due to doping Co and Mg in the HA structure, confirming the reduction of oxygen vacancies in HA nanoparticles and increasing HA crystal order because of Co and Mg dopants.^[^ ^60^ ^]^ This finding was also verified using high‐resolution XPS of O1s (Figure 3g–j). In the TFY mode of the O K‐edge XANES (Figure 4b), the intensities of features b and c were similar for all nanoparticles. Also, the pre‐edge peak (feature b) intensity in TFY was lower than in TEY mode. In XANES spectra, TFY and TEY modes were concerned with the bulk and surface of materials, respectively.^[^ ^61^ ^]^ As demonstrated in Figure 4a,b, the dominant changes in the O K‐edge XANES spectral features were seen in the TEY mode, indicating oxygen vacancies, especially in HA nanoparticles, dominated the surface compared to the bulk of nanoparticles. The O K‐edge XANES spectra of Co‐HA in TEY and TFY modes (Figure 4a,b) demonstrated a pre‐edge peak (feature a ≈530 eV) associated with the electron transitions to Co3d‐O2p hole states.^[^ ^62^ ^]^

The DOS diagram resulting in DFT calculation for the Co‐doped HA cell structure is shown in Figure 4d, exhibiting the states originated by the Co atom (blue spectrum) along with the total DOS of HA atoms (black spectrum) for up and down spins. As displayed in Figure 4d, the created states arose from the Co atom observed below the conduction band (featured as localized states). Figure S9 (Supporting Information) displays the DFT simulation results for the DOS diagram of the Mg‐doped HA cell structure and the states originated by the Mg atom (presented in the green spectrum). As shown in Figure S9 (Supporting Information), the created states in the conduction band arose from the Mg atom doping. Figure 4e shows the Ca L‐edge TFY XANES spectra of HA, Co‐HA, and Mg‐HA nanoparticles, and dominant peaks at ≈349.16 eV and ≈352.47 eV were related to the L_2_ and L_3_ absorption edges, respectively.^[^ ^59^ ^]^ The Ca^2+^ cation lacks 3d electrons, and 2p absorption arises from the transition from 2p^6^3d^0^ to 2p^5^3d^1^. Exchange interactions of electrons, crystal field, and spin‐orbit splitting dominate the L_2,3_ edge of 3d° cations. As demonstrated in Figure 4e, the L_2_ and L_3_ peaks of all samples exhibit nearly uniform intensity, and the pre‐edge peaks were observed in all samples. Pre‐edge peaks arose from crystal field splitting, originating from the geometric arrangement and symmetry of the atoms coordinating the Ca^2+^ ions.^[^ ^63^ ^]^ The influence of the crystal field on atomic multiplets results in forbidden lines in a spherically symmetric environment and rearranges the intensity across all spectral lines.^[^ ^63^ ^]^

The pre‐edge peaks were related to the symmetrical area around the Ca^2+^ ions. Principal pre‐edge peaks a_1_ (L_3_‐edge) and b_1_ (L_2_‐edge) were labeled in the Ca L‐edge XANES spectra by the analysis of de Groot et al.^[^ ^64^ ^]^ so that the splitting of a_1_ and a_2_ (as well as b_1_ and b_2_) was associated non‐linear dependence to the crystal field parameter values.^[^ ^64^ ^]^ According to deconvoluted spectra of Ca L_3_‐edge absorption in Figure 4e, the positions and full width at half maximum (FWHM) of the a_2_ and a_1_ peaks, the relative intensity of a_1_ to a_2_, as well as the energy difference between the a_2_ and a_1_ peaks (ΔL_3_), are given in Table S1 (Supporting Information). The shift of the a_1_ peak position, FWHM value, and ΔL_3_ were used to measure the order in the HA crystals.^[^ ^59^, ^63^, ^65^ ^]^ According to Table S1 (Supporting Information), by doping Co and Mg in the structure of HA nanoparticles, the pre‐edge peak a_1_ was shifted toward the lower energies, demonstrating that the nanoparticle's crystallinity increased because of Co and Mg dopants. Moreover, ΔL_3_ values in Table S1 (Supporting Information) demonstrated that the Mg‐HA sample had the largest energy split of 1.14 eV, indicating a highly crystalline sample. Also, a decrease in FWHM was observed for the a_1_ and a_2_ peaks by doping Co and Mg in the HA structure (Table S1, Supporting Information). Decreased peak widths in FWHM for the a_1_ and b_2_ peaks indicated a higher degree of phase order of HA nanoparticles because of Co and Mg dopants.^[^ ^63^ ^]^

Furthermore, the relative intensity of a_1_ to a_2_ indicated the magnitude of the crystal field.^[^ ^63^ ^]^ The relative intensity values of a_1_ to a_2_ in Table S1 (Supporting Information) suggested that the crystal field parameter in HA's crystal structure increased due to doping Co and Mg in HA nanoparticles. Figure S10 (Supporting Information) shows the 2D charge density difference (2D CDD) from the (001) plane of HA, Co‐doped HA, and Mg‐doped HA crystal cells. As observed in Figure S10 (Supporting Information), in the 2D CDD diagram of HA, charge depletion regions were around Ca and P atoms, and charge accumulation regions were around the oxygen atoms. Substituting a Co or Mg atom in place of one Ca atom in the HA cell raised the intensity of charge accumulation around the oxygen atoms neighboring the Co or Mg atom.

The Ca L‐edge XANES spectra of HA, Co‐HA, and Mg‐HA nanoparticles in TEY mode are displayed in Figure S11 (Supporting Information), and relevant parameters extracted from the deconvoluted Ca L_3_‐edge spectra (the positions and FWHM of the a_2_ and a_1_ peaks, ΔL_3_, and the relative intensity of a_1_ to a_2_) are presented in Table S2 (Supporting Information). By comparing TEY with TFY modes of Ca L‐edge XANES spectra, noticeable changes in the Ca L‐edge were observed, specially decrease in the crystal order at the surface of nanoparticles compared to the bulk, which was related to more oxygen vacancies and defects at the surface of nanoparticles.

Figure 4f displays the Co L‐edge XANES of the Co‐HA nanoparticles in TEY and TFY modes, including the Co L_3_‐edge (≈780.2 eV) and Co L_2_‐edge (≈795.1 eV), separated due to the 2p core‐hole spin‐orbit interaction.^[^ ^61^ ^]^ As exhibited in Figure 4f, the overall spectral line shape indicated characteristics with the line shape of the octahedral Co^2+^ ion, demonstrating Co dopants in cationic sites coordinated in an octahedral arrangement with oxygen ligands.^[^ ^61^ ^]^ By comparing TEY with TFY of Co L‐edge XANES spectra, two noticeable changes in the Co L_3_‐edge were seen with the risen low‐energy shoulders (features b and c) and the reduced feature a, demonstrating the generation of low‐coordinated octahedral Co^2+^ ions, verifying more oxygen vacancies at the surface of Co‐HA nanoparticles.^[^ ^61^, ^66^ ^]^ Upon comparing the TEY and TFY Co L‐edge XANES spectra, distinct alterations at the Co L_3_‐edge were observed: an increase in low‐energy shoulders (features b and c) and a decrease in feature a. These changes suggest the formation of low‐coordinated octahedral Co^2+^ ions, demonstrating increased oxygen vacancies at the surface of Co‐HA nanoparticles.

Mg K‐edge spectra of the Mg‐HA nanoparticles in TEY and TFY modes (Figure 4g) reflect spectral features B1, B2, and B3 centered at ≈1310, 1317, and 1328 eV, respectively, exhibiting substituting Mg^2+^ ions in the host cell structure.^[^ ^56^, ^63^ ^]^ By comparing TEY with TFY, two noticeable changes in the Mg K‐edge were observed with the reducing shoulders (features B1, B2, and B3) and the appearance of a pre‐edge peak (feature a ≈1308 eV). These changes indicated the generation of low‐coordinated octahedral Mg atoms at the surface of Mg‐HA nanoparticles due to more oxygen vacancies.^[^ ^60^, ^67^ ^]^

To further characterize the mineral phase of nanoparticles, the fourier‐transform infrared (FTIR) analysis was conducted to investigate the effect of Co and Mg dopants on the phosphate groups of HA. The FTIR spectra of all four samples are displayed in Figure S12 (Supporting Information). The vibrational bands observed at 477, 571, 604, 961, 1048, and 1089 cm^−1^ were attributed to PO_4_^3−^ groups,^[^ ^68^, ^69^ ^]^ while bands at 873, 1414, and 1456 cm^−1^ were associated with CO_3_^2−^ groups.^[^ ^68^, ^69^ ^]^ Furthermore, the bands at 3569 and 634 cm^−1^ corresponded to OH^−^ groups.^[^ ^70^ ^]^ There were no significant differences in the FTIR peak profiles, especially phosphate groups, which demonstrated that the mineral structure of HA was stable after doping with Co and Mg using the solid‐state reaction method. Figure S13 (Supporting Information) presents the deconvoluted FTIR spectra for all four samples, with green lines indicating the individual subbands in the 900–1200 cm^−1^ spectral region. The analysis revealed that accurately fitting the experimental data from all four samples requires the incorporation of eight subbands (green) corresponding to the phosphate group (ν_1_ and ν_3_ [PO_4_]^−3^ domains) of the HA molecular structure.^[^ ^71^ ^]^

### 2.3 Optical Properties and DFT Simulation

**Figure 5:**
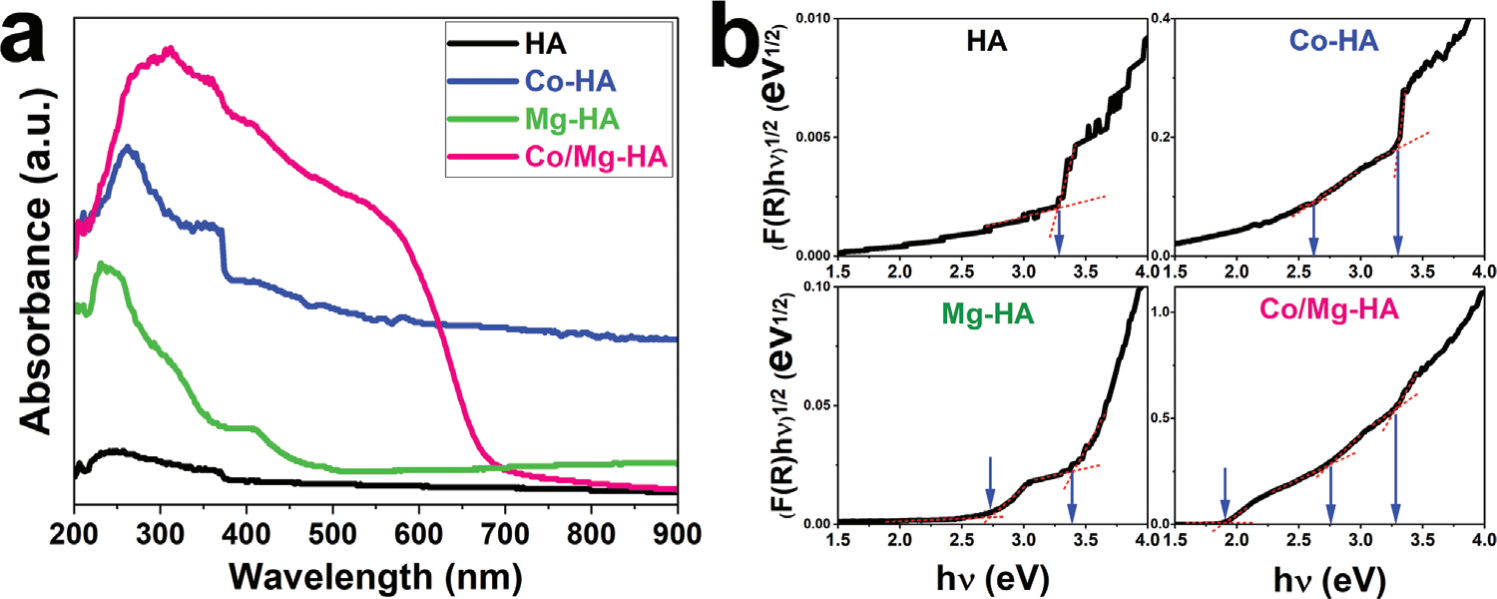
a) The absorbance UV‐Vis DRS spectra and b) the Tauc plots of HA, Co‐HA, Mg‐HA, and Co/Mg‐HA nanoparticles.

The ultraviolet‐visible diffuse reflectance spectra (UV–vis DRS) of HA, Co‐HA, Mg‐HA, and Co/Mg‐HA nanoparticles are displayed in **Figure** **5**a. The doped samples (especially the Co/Mg‐HA sample) had a significant absorption in the visible light and ultraviolet ranges compared to HA nanoparticles. The doping of Co and Mg elements increased the ability of HA nanoparticles to absorb visible light. By converting the absolute absorption measurements into the Kubelka‐Munk function,^[^ ^72^ ^]^ the band gap values of the samples were calculated. Using the curve of Kubelka‐Munk function versus photon energy, the band gap values of ≈3.26, 2.62, 2.74, and 1.94 eV for HA, Co‐HA, Mg‐HA, and Co/Mg‐HA were obtained, respectively (Figure 5b). A band gap of ≈3.26 eV indicated that the HA nanoparticles could generate photoexcited electron/hole pairs under visible light irradiation. Moreover, doping the HA with Co and Mg reduces its band gap and increases its photosensitivity in the visible light range.

The photoluminescence (PL) spectra of all four samples at an excitation wavelength of 267 nm are displayed in Figure S14 (Supporting Information). The emission spectrum at ≈408 nm mainly relates to the photo‐excited electron/hole recombination events between the valence and conduction bands or radiative recombination sites.^[^ ^73^ ^]^ According to the PL spectra in Figure S14 (Supporting Information), doping Co and Mg in the HA structure significantly reduced its fluorescence. The decrease in PL emission of the doped HA samples was attributed to new states in the band gap region due to Co and Mg dopants. The creation of new states caused by Co and Mg dopants in the electronic band structure of HA led to non‐radiative recombination sites. Subsequently, it reduced the intensity of PL emission.^[^ ^74^, ^75^ ^]^

**Figure 6:**
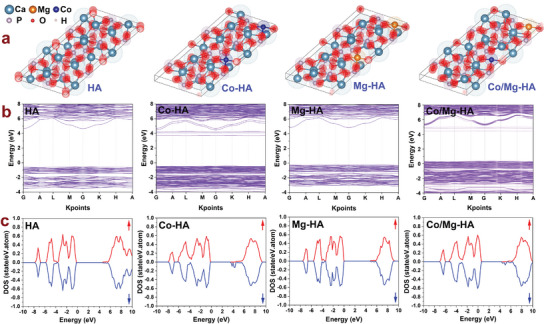
a) The modeled 3D atomic structures, b) electronic band structure, and c) DOS diagrams of HA, Co‐HA, Mg‐HA, and Co/Mg‐HA crystals.

**Figure** **6** shows the modeled 3D atomic structures (a), electronic band structures (b), and DOS diagrams (c) for HA, Co‐HA, Mg‐HA, and Co/Mg‐HA. According to the electronic structure of HA, its band gap value is 5.3 eV. Theoretical studies on the electronic structure of HA, as well as optical properties assessment of chemically synthesized HA nanoparticles, indicate that the band gap value of HA can be in the range of 4.86 to 5.23 eV and is considered an insulator material.^[^ ^19^, ^76^ ^]^ However, the band gap value for HA extracted from natural sources can be much lower than the 5.3 eV; defects in its atomic structure (e.g., oxygen vacancy) impact its electronic structure and, consequently, its band gap value.^[^ ^77^ ^]^ The XPS and XANES studies revealed the oxygen vacancies in the atomic structure of HA nanoparticles (Figure 3g and Figure 4a,b). Studying the electronic structure of HA revealed that oxygen vacancy led to the appearance of a state in the middle of its band gap. Figure S15a (Supporting Information) shows the 3D model of HA with oxygen vacancy (HA‐OV), (b) the electronic band structure, and (c) a DOS diagram of the HA‐OV crystal cell. As demonstrated in Figure S15b,c (Supporting Information), the vacancy in the atomic structure of HA led to changes in its electronic structure, shifting states in the band gap region and reducing its band gap from 5.3 to 3.3 eV. Furthermore, studying the influence of Mg doping on the electronic structure of HA indicated the emergence of new states above the valence band (Figure 6b). Meanwhile, Co doping in the HA structure led to the appearance of new states above the valence band, below the conduction band, and the localized states in the band gap region. The co‐doping of Co and Mg in the HA structure led to disturbance in the periodicity of the crystal and emerged as new states below the conduction band and above the valance band and localized states in the band gap region (Figure 6b).

**Figure 7:**
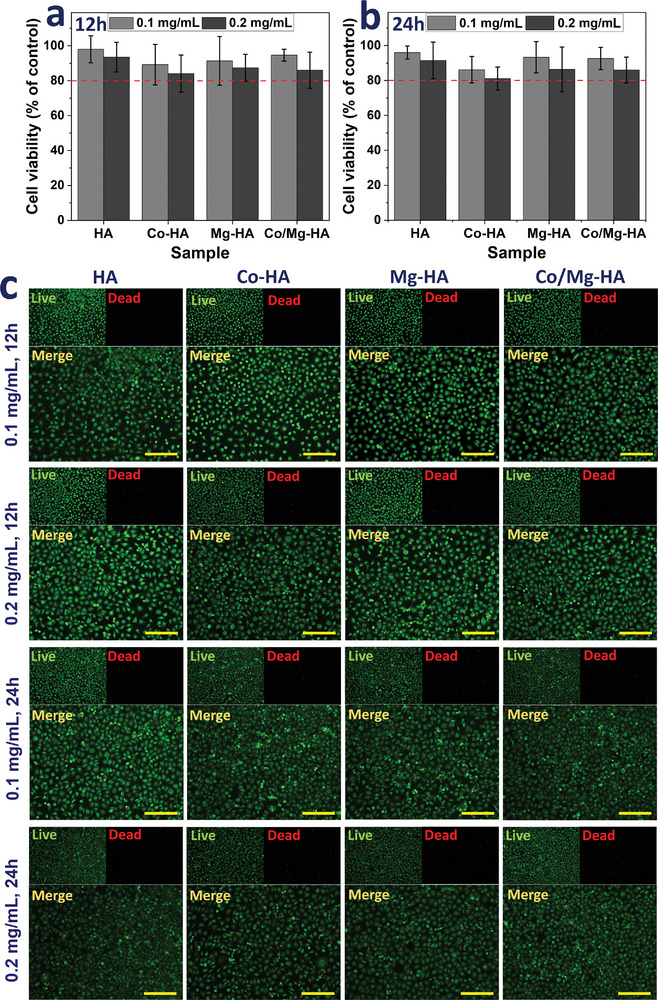
MTS graphs for culture treated by HA, Co‐HA, Mg‐HA, and Co/Mg‐HA nanoparticles at two concentrations of 0.1 and 0.2 mg mL^−1^ at a) 12 and b) 24 h, c) Live/Dead assay results for culture treated by HA, Co‐HA, Mg‐HA, and Co/Mg‐HA nanoparticles in two concentrations of 0.1 and 0.2 mg mL^−1^ in two‐time intervals of 12 and 24 h. (scale bar: 100 µm) (The data are presented as the mean ± SD).

Considerable modifications in the electronic structure of HA due to co‐doping of Co and Mg revealed a significant change in the optical properties of HA, such as a decrease in its band gap value. Also, according to the DOS diagrams (Figure 7c), HA's spin‐up and spin‐down states were symmetric, and Mg doping did not impact the symmetry of spins. However, HA's spin‐up and spin‐down states became asymmetric by Co doping, generating magnetization characteristics in the Co‐HA and Co/Mg‐HA nanoparticles. As displayed in Figure S16 (Supporting Information), the hysteresis loops of HA and Mg‐HA samples demonstrated negligible magnetic susceptibility, but Co‐HA and Co/Mg‐HA samples showed significant magnetic susceptibility. The saturation magnetic values for Co‐HA and Co/Mg‐HA samples were 7.14 and 4.92 emu g^−1^, respectively, while the respective values for HA and Mg‐HA samples were ≈0.04 emu g^−1^. Therefore, Co doping in the HA structure transformed it from a non‐magnetic material to a ferromagnetic material with meaningful magnetic susceptibility.

To provide a comprehensive understanding of the impacts of Co and Mg dopants in the HA cell structure, the contributions of orbitals and their energies on the projected density of states (PDOS) are displayed in Figure S17 (Supporting Information). The O (s, p orbitals), P (s, p orbitals), and Ca (s, p orbitals) states were affected upon introducing Co and Mg atoms into the HA crystal. The delocalization of PDOS of O, P, and Ca (s, p orbitals) for the doped samples exhibited hybridization of the Co and Mg orbitals with the O, P, and Ca orbitals of HA. Interestingly, the Co atom led to spin polarization of HA's O, P, and Ca orbitals. Also, spin‐polarization in Co‐HA and Co/Mg‐HA systems predominantly originated from the d orbitals of Co (Figure S17b,d, Supporting Information).

### 2.4 Biological Assessments

**Figure** **7**a,b show the MTS graphs for culture exposed samples at two concentrations of 0.1 and 0.2 mg mL^−1^ at 12 and 24 h, respectively. As displayed in Figure 7a,b, cell viability is greater than 80% in all samples at all concentrations and times, demonstrating biocompatibility, and that all tested samples were harmless to fibroblast cells. The cytotoxicity of the samples was also assessed using the Alamar Blue assay to evaluate cell proliferation and viability in response to nanoparticle exposure. As shown in Figure S18a,b (Supporting Information), cell viability exceeded 80% for the culture exposed samples at two concentrations of 0.1 and 0.2 mg mL^−1^ at 12 and 24 h, respectively, consistent with the results observed in the MTS assay. Figure 7c displays the LIVE/DEAD assay results for culture treated nanoparticles. As demonstrated in the fluorescence microscopic images in Figure 7c, nanoparticles did not affect cell viability. Also, fibroblast cells with their typical elongated morphology (green color) were well adhered and spread, and only a few dead cells (red color) were observed in the exposed cells.

Figure S19 (Supporting Information) displays the hemolysis percentages for the four samples at concentrations of 0.1 and 0.2 mg mL^−1^. The HA sample exhibited below 5% hemolysis, classifying it as a highly hemocompatible biomaterial according to the ASTM F 756‐00 standards.^[^ ^78^ ^]^ The doped samples demonstrated even lower hemolysis compared to the HA sample. Notably, doping HA nanoparticles with Co and Mg did not negatively impact hemolysis; instead, it enhanced the blood compatibility of the nanoparticles, especially the Co‐HA sample.

**Figure 8:**
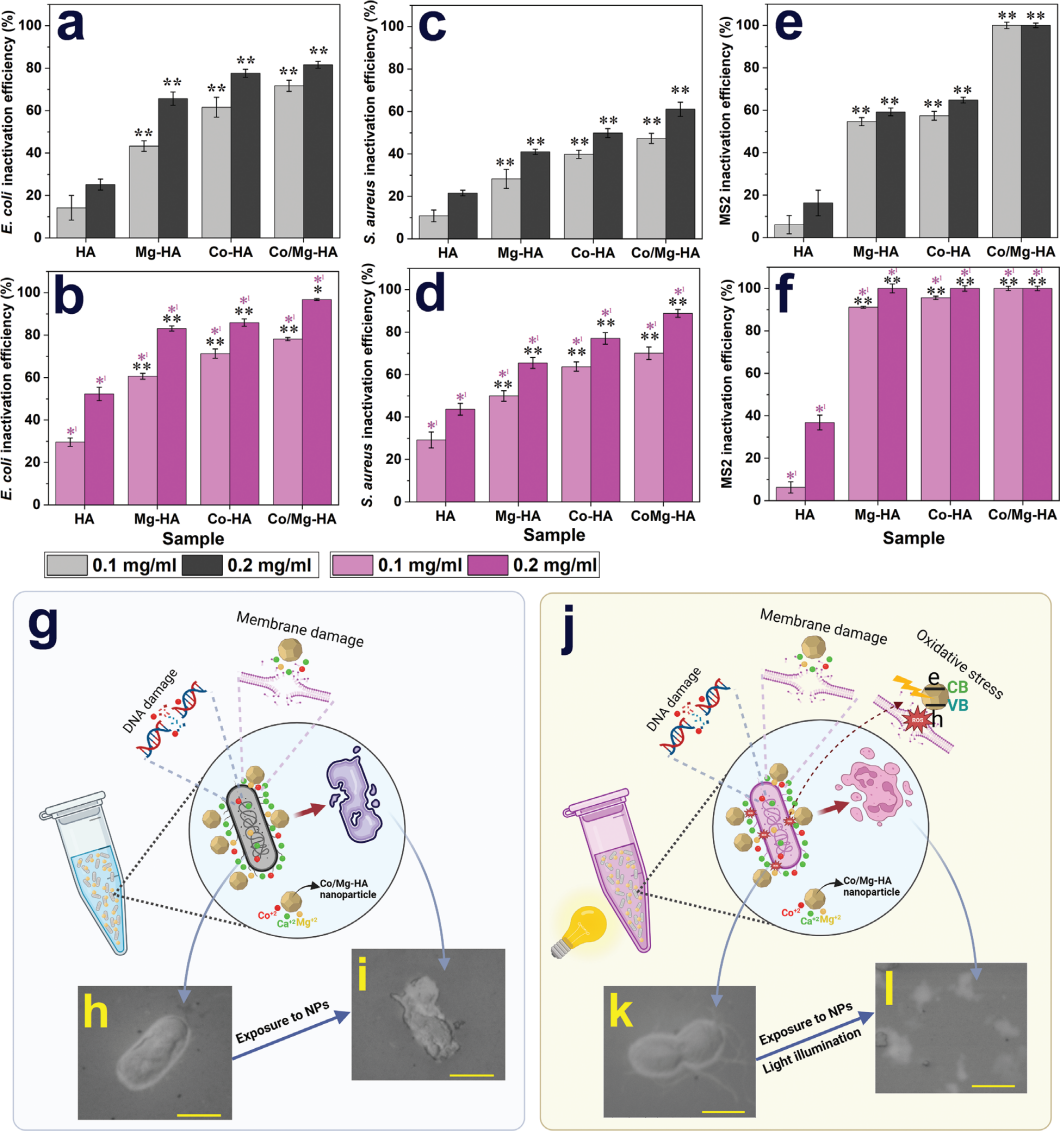
Antimicrobial activity results of HA, Co‐HA, Mg‐HA, and Co/Mg‐HA nanoparticles under a,c,e) dark and b,d,f) visible light irradiation against a,b) *E. coli* and c,d) *S. aureus* bacteria and e,f) bacteriophages MS2, schematic of the mechanisms of antibacterial activity of Co/Mg‐HA nanoparticles under e) darkness and h) light illumination, SEM images from *E. coli* after 12 h incubation f) without and g) with exposure to Co/Mg‐HA nanoparticles SEM images of *E coli* bacteria i) without and j) with exposure to Co/Mg‐HA nanoparticles under visible light irradiation. (scale bar: 1 µm) (The data are presented as the mean ± SD. *p < 0.05 and **p < 0.01.).

The antimicrobial activity results for the four nanoparticles against *E. coli* (Gram‐negative) and *S. aureus* (Gram‐positive) bacteria and bacteriophages MS2 under dark and visible light irradiation are depicted in **Figure** **8**. As illustrated in Figure 8a–c, the HA nanoparticles have antibacterial activity, and doping with Co and Mg increased their antibacterial activity more than 2.5‐ and 4‐fold, respectively, for *E. coli* and *S. aureus* bacteria at 0.1 mg mL^−1^ concentrations. Also, the superior antibacterial behavior was observed in the Co/Mg‐HA sample. Furthermore, it is worth noting that all nanoparticles showed better antibacterial activity under visible light illumination (Figure 8b–d).

There are known mechanisms for the antibacterial activity of HA or metal oxide nanoparticles.^[^ ^79^, ^80^, ^81^, ^82^ ^]^ One of the mechanisms involves the electrostatic interactions between the cell membrane and nanoparticles caused by the charge difference between the bacterial membrane and the nanoparticles.^[^ ^79^, ^80^ ^]^ Polysaccharides in the bacterial membrane and metal cations on the surface of HA nanoparticles interact via electrostatic forces.^[^ ^80^ ^]^ The nanoparticle accumulation on the cell surface changes the bacteria membrane's structure and permeability and can lead to the release of membrane proteins and intracellular factors.^[^ ^81^ ^]^ The loss of bacterial homeostasis by metal ions released from HA nanoparticles is another reason for the antibacterial activity of nanoparticles.^[^ ^82^, ^83^ ^]^ Since metabolic functions in bacteria are regulated with the help of enzymes, the survival of bacteria depends on the balance of metal elements, and the changes in the ions' concentration disrupt the metabolic function of bacteria.^[^ ^84^ ^]^

Mg^2+^ ions released from Mg‐doped HA nanoparticles lead to osmotic stress on the bacterial cell wall and can also disrupt bacteria growth.^[^ ^85^ ^]^ Xie et al.^[^ ^86^ ^]^ demonstrated that Mg^2+^ ions are effective antibacterial agents against *S. aureus*, disrupting their membrane and killing the bacteria. The mixture of Ca^2+^ and Mg^2+^ ions in the culture stimulates the inhibitory effect of *E. coli* and *S. aureus*.^[^ ^85^, ^87^ ^]^ Indeed, the co‐presence of Ca^2+^ and Mg^2+^ ions in the culture can create an effective antimicrobial agent to prevent pathogens' growth.^[^ ^88^ ^]^ Also, Co^2+^ ions have antibacterial properties and induce antibacterial activity of Co‐HA.^[^ ^89^, ^90^, ^91^ ^]^ Co^2+^ ions released from nanoparticles can bind to bacterial DNA, which may affect the processes of replication, repair, and transcription.^[^ ^92^ ^]^ Direct binding of Co^2+^ ions to DNA can change DNA topology, which roadblocks the function of DNA binding proteins.^[^ ^92^ ^]^ In the meantime, Co^2+^ ions specifically affect bacterial DNA polymerization, causing DNA damage.^[^ ^93^ ^]^ Moreover, Co^2+^ ions hinder the response of SOS‐dependent repair pathways and DNA repair by harming RecBCD function, affecting the bacterium's survival.^[^ ^92^ ^]^ Also, Co^2+^ ions lead to acidic physiological conditions, permitting Co^2+^ to exert their toxicity by interacting with the DNA and DNA metabolizing enzymes.^[^ ^94^ ^]^ More interestingly, the synergistic cytotoxicity of Co^2+^ and Mg^2+^ for bacteria exhibits that they can aggravate the deactivation of pathogens.

Figure 8e,f depicts the antiviral activity results for the four nanoparticles against bacteriophages MS2 under dark and visible light irradiation. As illustrated in Figure 8e, the HA nanoparticles demonstrated feeble antiviral behavior, and doping with Co and Mg raised their antiviral activity more than 10‐fold at 0.1 mg mL^−1^ concentration under dark. More interestingly, the Co/Mg‐HA sample inactivated 100% of bacteriophages MS2 at 0.1 mg mL^−1^ concentration. Light illumination increased the antiviral activity of Co‐ and Mg‐doped HA samples from 55% to 100% at 0.2 mg mL^−1^ concentration (Figure 8e,f).

Tang et al.^[^ ^95^ ^]^ demonstrated that the MS2 virus in particle (inorganic/organic) containing solutions with Ca^+2^ ions led to its deactivation. Motoike et al.^[^ ^96^ ^]^ found that the coexistence of Ca and Mg in dolomite particles provides an advantage for the antiviral effect against the influenza virus. Research studies on metal‐dependent enzymes and proteins for viruses revealed that ions such as Ca^+2^, Co^+2^, and Mg^+2^ can influence ligand coordination sets and geometry.^[^ ^97^, ^98^ ^]^ Interacting viral proteins and nucleoproteins with metal cations in the surrounding fluids of viral can lead to viral inactivation.^[^ ^97^, ^98^ ^]^

Figure 8e displays a schematic of the mechanisms of antibacterial activity of Co/Mg‐HA nanoparticles under darkness. Also, the SEM images from *E. coli* after 12 h incubation without and with exposure to Co/Mg‐HA nanoparticles are displayed in Figure 8f and g, respectively. As shown in Figure 8g, the cell structure of *E. coli* bacteria was harshly damaged and deformed after exposure to Co/Mg‐HA nanoparticles.

Since HA has been used as a well‐known and practical material in the structure of implants/scaffolds, its atomic structure can be modified/enhanced by using dopants while maintaining its biocompatibility. Thus, it can also prevent infection after implanting in the human body.^[^ ^99^, ^100^ ^]^ Furthermore, modifying the HA structure with purposeful dopants, which can have several functions, can provide some desirable properties simultaneously.^[^ ^100^ ^]^ The Co^2+^ and Mg^2+^ ions are accelerating factors (angiogenic and osteogenic) in regenerating new bone tissue.^[^ ^101^, ^102^ ^]^ Also, Co^2+^ and Mg^2+^ ions have antibacterial effects, and simultaneously, using both elements in HA's structure can synergistically boost its antibacterial activity.

Our investigations on the optical properties of HA extracted from the cow bone showed sensitivity to visible light, and Co/Mg doping raised its photosensitivity significantly. The photosensitivity of natural HA nanoparticles causes the generation of photo‐excited electron‐hole pairs under visible light illumination and subsequently provides the reactive oxidative species (ROS) on the surface of nanoparticles.^[^ ^103^ ^]^ When nanoparticles are exposed to radiation, ROS are produced on the surface of nanoparticles.^[^ ^103^, ^104^ ^]^

The ROS created by photo‐excited electron/hole pairs are harmful to damage and destroy cell membranes and intracellular materials.^[^ ^21^, ^103^ ^]^ Figure 8h shows a schematic of the interaction of Co/Mg‐HA nanoparticles under light illumination. As depicted in Figure 8h, ROS can react with the cell membrane and proteins of bacteria and destroy the bacteria. Figure 8i,j show the SEM images of *E coli* bacteria (i) without and (j) with exposure to Co/Mg‐HA nanoparticles under visible light irradiation conditions. As shown in Figure 8j, the structure of *E coli* bacteria collapsed after 12 h of exposure to Co/Mg‐HA nanoparticles under light illumination.

The photosensitivity property of doped/modified natural HA nanoparticles makes it possible for implants/scaffolds to be sterilized under visible light irradiation. In addition, the characteristics of light sensitivity and the biocompatibility of nanoparticles can promise broader applications of these nanoparticles in nano‐biotechnology, such as using them for antibacterial surfaces or disinfecting contaminated water. It is worth noting that due to nanoparticles' magnetic properties, they can be separated/removed after purifying the environment using an external magnetic field.

## 3 Conclusions

This research involved the extraction of HA particles from cow bone biowaste, followed by the synthesis of Co/Mg co‐doped natural HA using the solid‐state reaction technique. The resulting Co/Mg co‐doped natural HA nanoparticles exhibited a high level of crystallinity (99%) and contained quantum dot crystals. It was observed that the HA nanoparticles produced through the high‐energy ball milling process had significant oxygen vacancies on their surface, contributing to their band gap falling within the visible light range (3.26 eV). By introducing Co and Mg dopants, the band gap of the natural HA nanoparticles decreased from 3.26 to 1.94 eV due to the creation of new states within the band gap.

Moreover, the antibacterial properties of the natural HA nanoparticles were enhanced when exposed to visible light, showing a twofold increase in activity. The incorporation of Co, Mg, and Co/Mg into the HA nanoparticles resulted in a significant improvement in antibacterial performance – with increases of more than 2.5‐fold, 4‐fold, and 4.5‐fold under dark conditions, and 2‐fold, 2.3‐fold, and 2.7‐fold under visible light exposure, respectively. Furthermore, MTS assays and LIVE/DEAD assays conducted on the natural HA, Co‐HA, Mg‐HA, and Co/Mg‐HA nanoparticles demonstrated their biocompatibility with cells. The Co/Mg‐HA nanoparticles, with their ferromagnetic properties, sensitivity to visible light, cytocompatibility, and antibacterial effects, were identified as a potentially superior material for biomedical applications requiring anti‐infection capabilities.

## 4 Experimental Section

### Materials and Solid‐State Reaction Process

HA powder was derived from bovine bone biowaste. For this purpose, after burning the cortical bone of the cow using an oxyacetylene flame (400 °C), it was subjected to heat treatment in a furnace at 820 °C for 4 h to obtain white HA powder. The HEBM process was performed by a planetary ball mill (RETSCH‐PM 400, Germany) to grind HA, Co‐HA, Mg‐HA, and Co/Mg‐HA powders. The tungsten carbide cups were filled with HA, HA and Co powders (99.8%, <2 µm, Sigma–Aldrich), HA and Mg powders (99.8%, <3 µm, Merck, Germany), and HA and Co/Mg (0.5/0.5) powders with the molar ratio of 9 (HA):1 (metal). The ball milling process was conducted in an argon atmosphere at 450 rpm, with a ball‐to‐powder ratio of 12:1, for 24 h.

### Characterization

A TEM (FE‐TEM, Tecnai F20, 200 kV) was used to study the morphology of powders. Also, high‐angle annular dark‐field scanning TEM (HAADF‐STEM) images were obtained on a STEM.

The synchrotron SAXS and WAXS examinations were performed in the beam‐line BL11‐NCD of the ALBA synchrotron light source facility in Spain. Experimentations were conducted at ambient temperature with a beam stop size of 4 mm, a sample‐detector distance of 7573 mm, and a q range of 0.1933 to 6.030 Å^−1^. The wavelength and spot size of the monochromatic X‐ray beam were 1.24 Å and 25 µm × 25 µm, respectively.

The XANES studies were conducted in the beam‐line PIRX of the SOLARIS synchrotron radiation center in Poland. The measurements were conducted in ultrahigh vacuum conditions (7 × 10^−10^ Torr). During the measurements, a monochromatic radiation beam was directed toward the sample surface at a 45° angle, with a focused spot size of 0.1 × 0.2 mm^2^. The spectra were obtained in both total fluorescence yield (TFY) and total electron yield (TEY) modes.

The XPS measurements were conducted to analyze the bonding characteristics of nanoparticles using a PHl 5000 VersaProbe Scanning ESCA Microprobe instrument (ULVAC‐PHI; Chigasaki Japan).

The FTIR spectrometer (Vertex 80v FT‐IR spectrometer Bruker Inc., USA) was utilized to determine chemical bonds in the nanoparticles.

The UV–vis DRS spectra of samples were performed over the wavelength range of 250–900 nm using a Jasco V‐570 spectrophotometer.

The PL emission spectra of the nanoparticles were conducted using an Agilent Cary Eclipse fluorescence spectrophotometer utilizing an excitation source with a 270 nm excitation wavelength.

The magnetic measurements (M‐H loops) of samples were studied using a vibrating sample magnetometer (VSM LakeShore 7410 magnetometer).

To study the exothermic reaction between the HA and metal powders, DSC measurements were performed using a Mettler‐ToledoTGA/DSC 3+. The samples were heated from 25 to 1000 °C with a heating rate of 10 °C min^−1^.

### Biological Assessments

*Cytocompatibility*: The NIH‐cultured 10T1/2 cells (model cells line, obtained from mouse embryo fibroblasts) at 2 × 10^4^ cells cm^2^ cell seeded on a plate cell culture dish and incubated for 8 h for cell attachment to the plate surface. Then, the cell culture medium was changed to media supplemented with different concentrations of studied nanoparticles. For MTS assays, after indicated times of incubation at 12 and 24 h with the respective nanoparticles, an equivalent amount of MTS substrate to culture media (1:10 v:v) was added to the cells, followed by further incubation for 4 h at 37 °C and 5% CO_2_. The absorbance intensity was determined at 450 nm using a Fluostar OPTIMA spectrophotometric plate reader (BMG Lab Technologies, Germany). Besides, the absorbance of samples treated with Alamar Blue reagent was measured at 570 nm (reduced form; resorufin) and 620 nm (oxidized form; resazurin) after 4 h incubation. The results were quantified by determining the percentage reduction of Alamar Blue, which was then expressed relative to the control group (untreated cells), as previously reported.^[^ ^105^ ^]^

The LIVE/DEAD cell viability assay kit, encompassing ethidium homodimer and calcein acetomethoxy ester (calcein AM), was obtained from Invitrogen, and the assay was conducted following the manufacturer's prescribed protocol. The samples were washed with 4‐(2‐Hydroxyethyl)piperazine‐1‐ethanesulfonic acid (HEPES) and treated with calcein (0.5 µL mL^−1^, to stain the alive cells green) and ethidium homodimer (2 µL mL^−1^, to stain the dead cells red) in HEPES. Optical images were acquired using a fluorescent microscope (Leica TCS SP8) with a 10x objective.

*Hemolysis assay*: Blood samples (5 mL) were collected from male Wistar rats and immediately drawn into K2‐EDTA‐coated Vacutainer tubes to prevent coagulation. The samples were centrifuged at 1000 RCF for 10 min to separate intact red blood cells (RBCs) from the supernatant.

The plasma and buffy coat were gently aspirated using a micropipette. Sterile 150 mM NaCl was added to restore the original plasma volume, followed by gentle mixing and subsequent centrifugation. This washing procedure was repeated three times. Following the final centrifugation, the supernatant was aspirated, and the remaining RBCs were diluted in PBS at pH 7.4 to achieve a 2% (v/v) RBC suspension. Then, the RBC suspension was aliquoted into multiple test tubes, and an equal volume of nanoparticle suspensions was added at a 1:1 (v/v) ratio. After incubation of samples at 37 °C for 2 h, they were centrifuged at 1000 RCF for 10 min to pellet the intact erythrocytes.

Additionally, RBC suspensions were mixed with distilled water and pure PBS to serve as positive and negative controls, respectively. A multichannel pipette was used to transfer 200 µL of supernatant from each well into a clear, flat‐bottomed 96‐well plate. The absorbance of the supernatants was then measured at 540 nm using a UV–vis spectrophotometer, and the percentage of hemolysis was calculated using the following formula:^[^ ^106^ ^]^

(1) $$\begin{array}{ccc} & & \mathrm{Hemolysis}\;\left(\%\right)\\ & & \;\;\;=[(\mathrm{Absorbance}\;\mathrm{of}\;\mathrm{Sample}\;-\;\mathrm{Absorbance}\;\mathrm{of}\;\mathrm{Negative}\;\mathrm{Control})/\\ & & \;\;\;(\mathrm{Absorbance}\;\mathrm{of}\;\mathrm{Positive}\;\mathrm{Control}\;-\;\mathrm{Absorbance}\;\mathrm{of}\;\mathrm{Negative}\;\mathrm{Control})]\\ & & \;\;\;\times \;100 \end{array}$$

*Antibacterial Activity*: To study the antibacterial efficacy of the nanoparticles, they were evaluated against *E. coli* BL21 (Gram‐negative) and *S. aureus* DSM 105 252 (Gram‐positive). Each bacterial strain was exposed to the nanoparticles individually for 12 h under both dark and light (illuminated by white LED light, 5 W) conditions. Subsequently, alterations in bacterial concentrations were quantified via sample plating. The initial concentration of *E. coli* and *S. aureus* cells was set at 10^4^ colony‐forming units per milliliter (CFU/mL). These experiments were conducted in biological triplicates at a controlled temperature of 37 °C and an agitation rate of 350 rpm.

For electron microscopy images, samples of 10^6^ CFU mL^−1^ of *E. coli* cultures after exposing to the Co/Mg‐HA nanoparticles for 12 h under both dark and light were centrifuged at 4400 g for 10 mins and resuspended in phosphate‐buffered saline (PBS) (control sample) or Co/Mg‐HA nanoparticles dispersions in PBS (0.2 mg mL^−1^) solution. The sample was then dropped on a silicon wafer and allowed to dry. It was then washed thrice with PBS, 50% PBS, and deionized water.

*Antiviral Assay*: The antiviral activity of the nanoparticles was assessed using bacteriophages MS2 (as a good surrogate for studies of viruses infecting eukaryotic cells)^[^ ^107^ ^]^ by a methodology previously reported.^[^ ^108^ ^]^ Briefly, Petri plates were first prepared with 20 mL of LB‐agar medium. Then, 4 mL of top LB agar mixed with 200 µL of refreshed *E. coli* (C3000) culture was poured onto the plates.

MS2 (initial titer ≈10^7^ PFU mL^−1^) was exposed to nanoparticles (0.1 – 0.2 mg mL^−1^) for 6 h at room temperature under both dark and light conditions. Then, appropriate dilutions of the phage suspension exposed to the nanoparticles were prepared. Eight 5 µL droplets of each diluted sample were placed onto the top agar layer. Following a 24 h incubation at 37 °C, the plaques were counted and recalculated as plaque‐forming units per milliliter (PFU/mL).

### DFT Simulation

The spin‐polarized DFT calculations were performed by VASP 6.3.0 using the Perdew‐Burke‐Ernzerhof (PBE) exchange‐correlation function. Atomic relaxation was performed using a conjugate gradient algorithm until the total free energies became less than 10^−3^ eV, and the break energy for the electronic loop was 10^−4^ eV. The plane‐wave basis assigned had an energy cutoff of 560 eV, and partial occupancies were defined using a Gaussian smearing width of 0.05 eV. A Γ‐centered mesh density of 0.03/Å was used to sample the Brillouin zone. The interactions between the atomic cores and electrons resembled using the projector‐augmented wave (PAW) pseudopotentials. A primitive cell with 44 atoms (Ca_10_(PO_4_)_6_(OH)_2_) was used for calculations. To simulate the Co‐ and Mg‐doped HA, one Ca atom was replaced by a Co or Mg atom or a combination of Co and Mg (co‐doped). For the co‐doped sample, a 2×1×1 supercell was used. K‐path for band structure calculations was sampled using the Sumo toolkit.^[^ ^109^ ^]^ The “easyunfold” package was employed to unfold the band structure of the supercell.^[^ ^110^ ^]^

### Statistical Analysis

All experimental results were analyzed using Microsoft Office 2016 and Origin 2021b software. Data were expressed as mean ± SD. One‐way ANOVA and Student's t‐test were used to determine statistical significance. Data analysis was considered statistically significant when *p* <0.05.

## Conflict of Interest

The authors declare no conflict of interest.

## Supporting information



Supporting Information

## Data Availability

The data that support the findings of this study are available on request from the corresponding author. The data are not publicly available due to privacy or ethical restrictions.
