# Supplementary material for: Visible Light‐Sensitive Sustainable Quantum Dot Crystals of Co/Mg Doped Natural Hydroxyapatite Possessing Antimicrobial Activity and Biocompatibility
Source: Small. 2024 Oct 24;20(52):2405708. doi: 10.1002/smll.202405708 (PMC11673459; doi:10.1002/smll.202405708)
Supplement: Supplementary file 1 — Supporting Information [file SMLL-20-2405708-s001.docx]

**Supporting Information**

Visible light-sensitive sustainable quantum dot crystals of Co/Mg doped natural hydroxyapatite possessing antimicrobial activity and biocompatibility

Hossein Maleki-Ghaleh^1,^^[[1]](#footnote-1)^*, Bartosz Kamiński^1^, Ehsan Moradpur-Tari^2^, Sada Raza^1^, Mehdi Khanmohammadi^3^, Rafał Zbonikowski^1^, Mohammad Sadegh Shakeri^4^, M. Hossein Siadati^5^, Ali Akbari-Fakhrabadi^6^, Jan Paczesny^1,^^[[2]](#footnote-2)^**

^1^ Institute of Physical Chemistry, Polish Academy of Sciences, Kasprzaka 44/52, 01-224 Warsaw, Poland

^2^ Institute of Technology, University of Tartu, Nooruse 1, 50411 Tartu, Estonia

^3^ Biomaterials Group, Materials Design Division, Faculty of Materials Science and Engineering, Warsaw University of Technology, Wołoska 141, Warsaw 02-507, Poland

^4^ Institute of Nuclear Physics Polish Academy of Sciences, PL-31342, Krakow, Poland

^5^ Materials Science and Engineering Faculty, K. N. Toosi University of Technology, Tehran, Iran

^6^ Advanced Materials Laboratory, Department of Mechanical Engineering, University of Chile, Santiago, 8370456, Chile


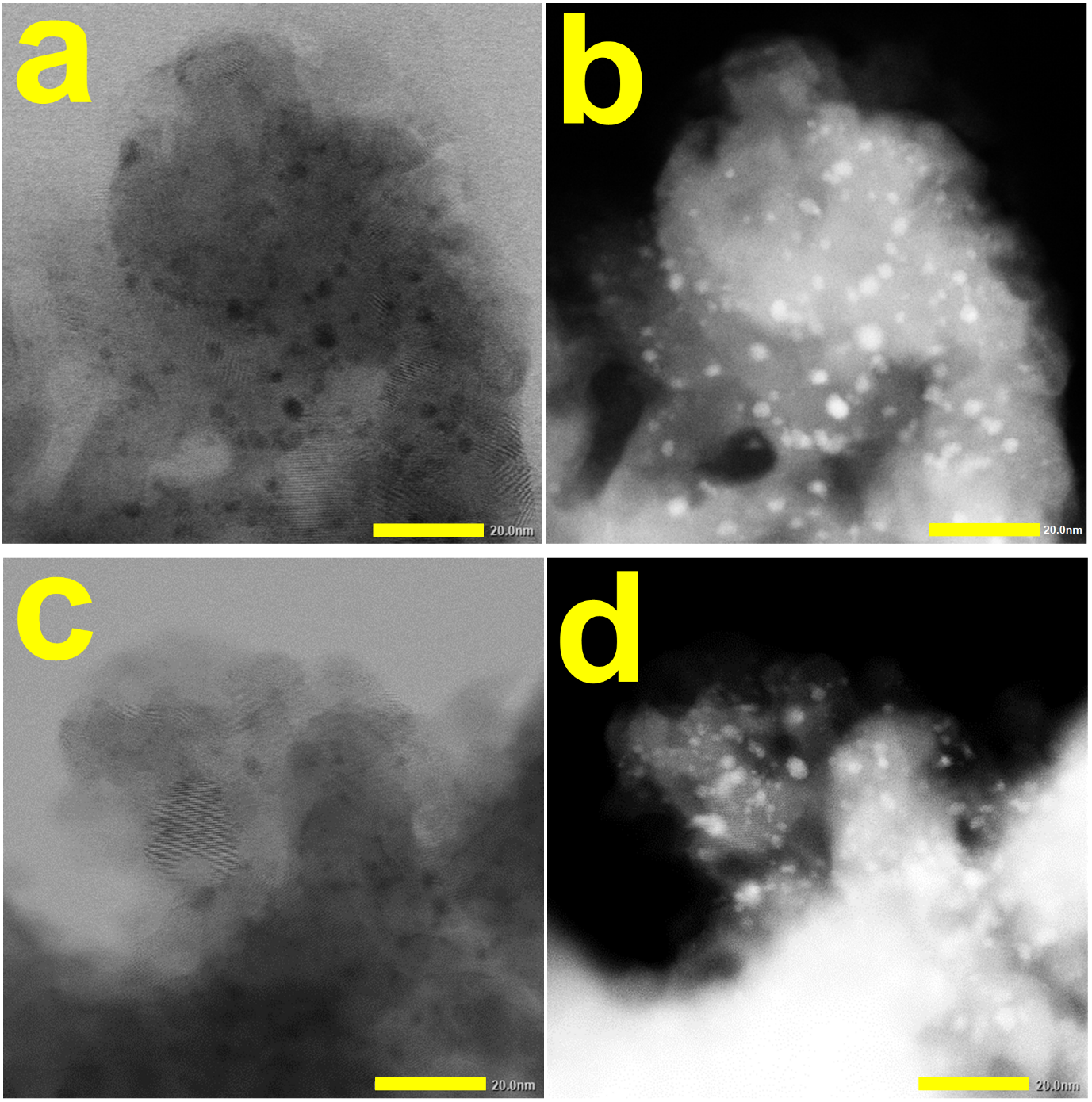


Figure S1. (a,c) bright-field (BF) TEM and (b,d) high-angle annular dark-field scanning TEM (HAADF-STEM) images of HA (a,b) and (c,d) Co/Mg-HA nanoparticles.


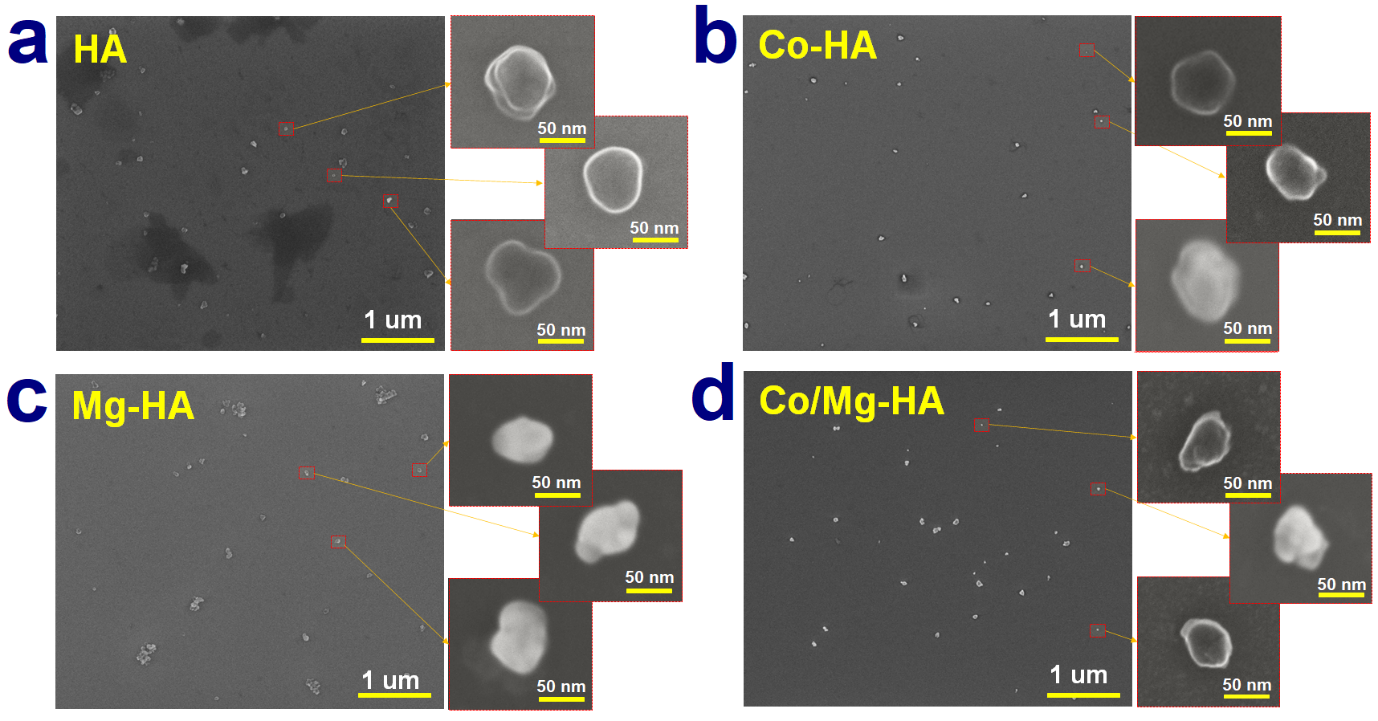
Figure S2. SEM images of HA (a), Co-HA (b), Mg-HA (c), and Co/Mg-HA (d) nanoparticles.


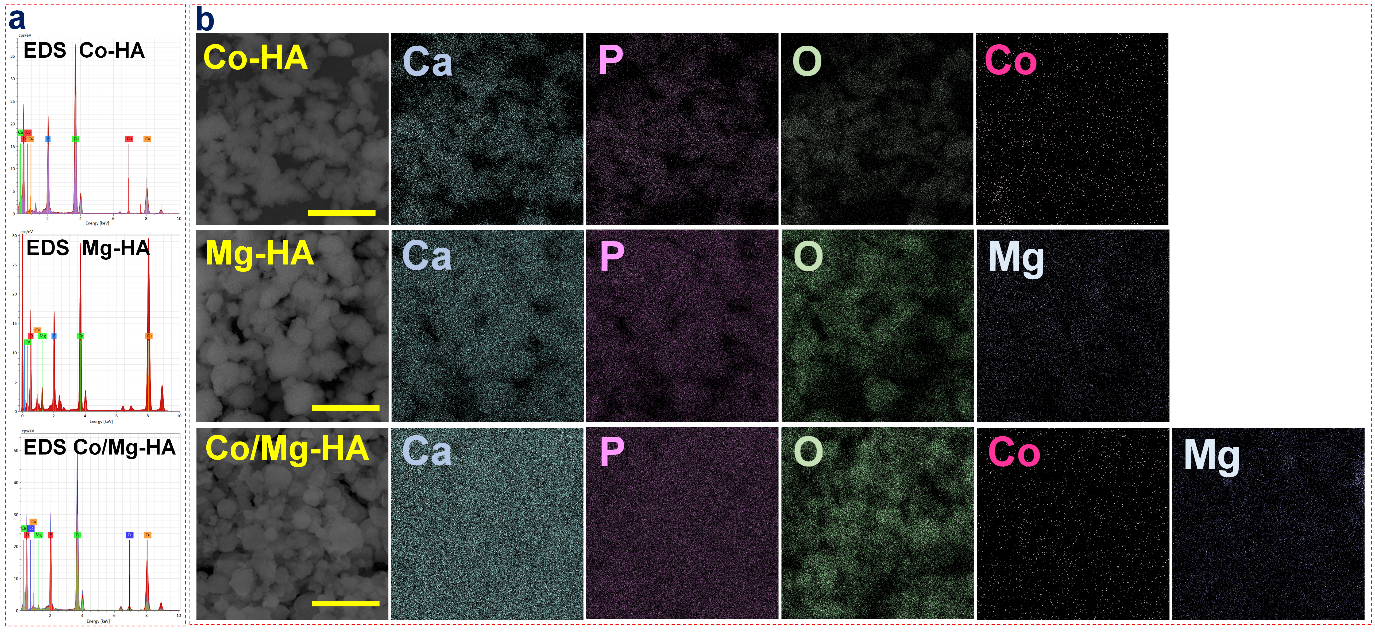


Figure S3. a) EDS elemental analysis and b) EDS-MAP elemental analysis of Co-HA, Mg-HA, and Co/Mg-HA nanoparticles. (scale bar: 2 µm)


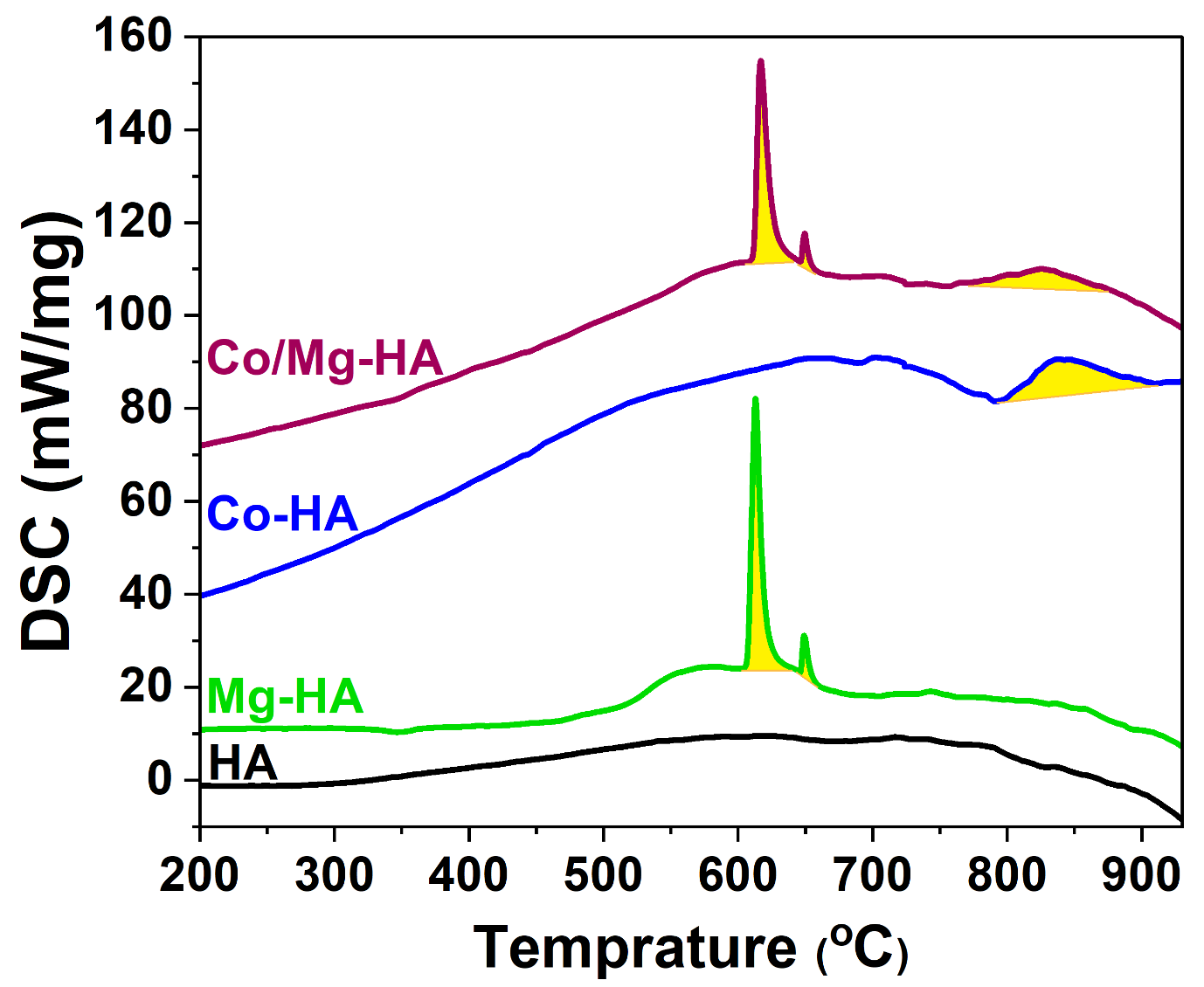


Figure S4. DSC curves of HA, Mg-HA, Co-HA, and Co/Mg-HA samples.


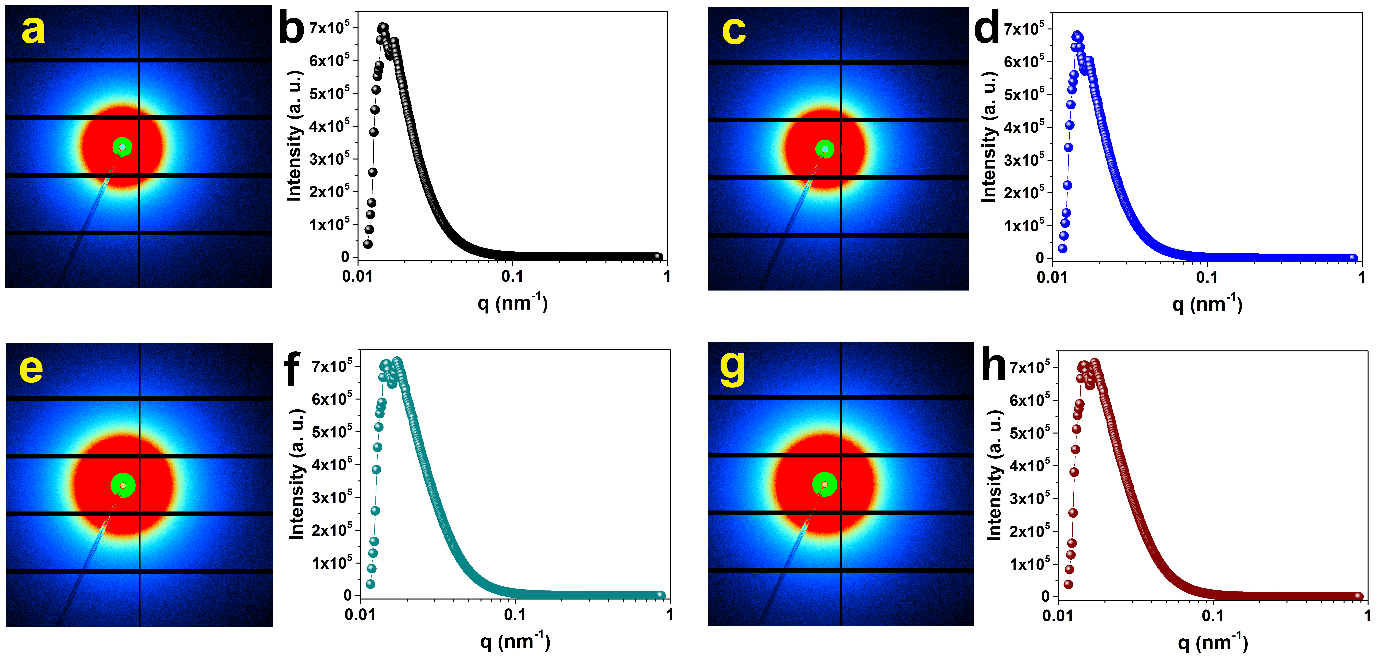


Figure S5. 2D SAXS diffraction patterns of a) HA, c) Co-HA, e) Mg-HA, and g) Co/Mg-HA nanoparticles, 1D SAXS diffraction patterns of b) HA, d) Co-HA, f) Mg-HA, and h) Co/Mg-HA nanoparticles.


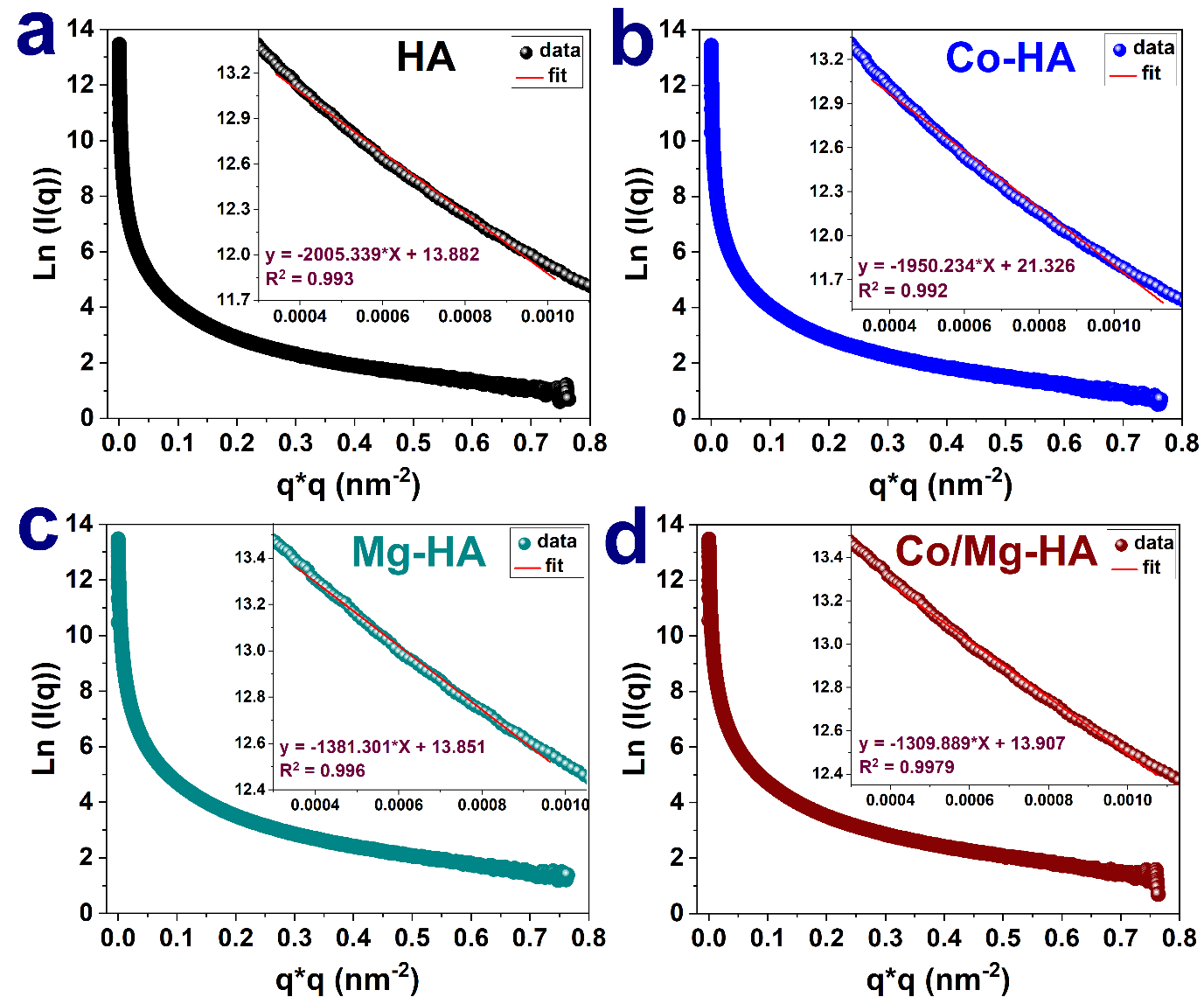


Figure S6. q^2^-LnI diagrams of HA, Mg-HA, Co-HA, and Co/Mg-HA nanoparticles.


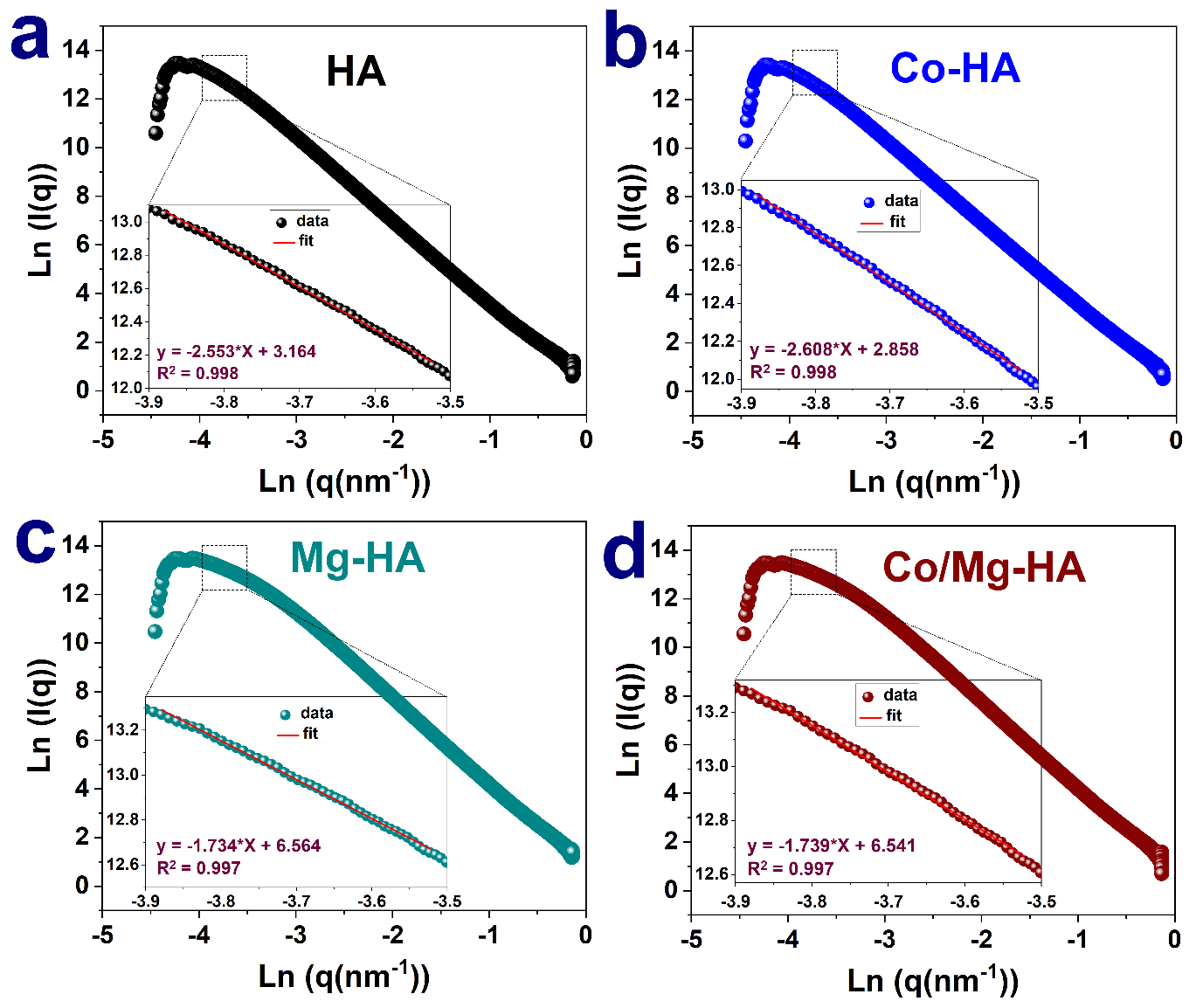


Figure S7. Lnq-LnI diagrams of HA, Mg-HA, Co-HA, and Co/Mg-HA nanoparticles.


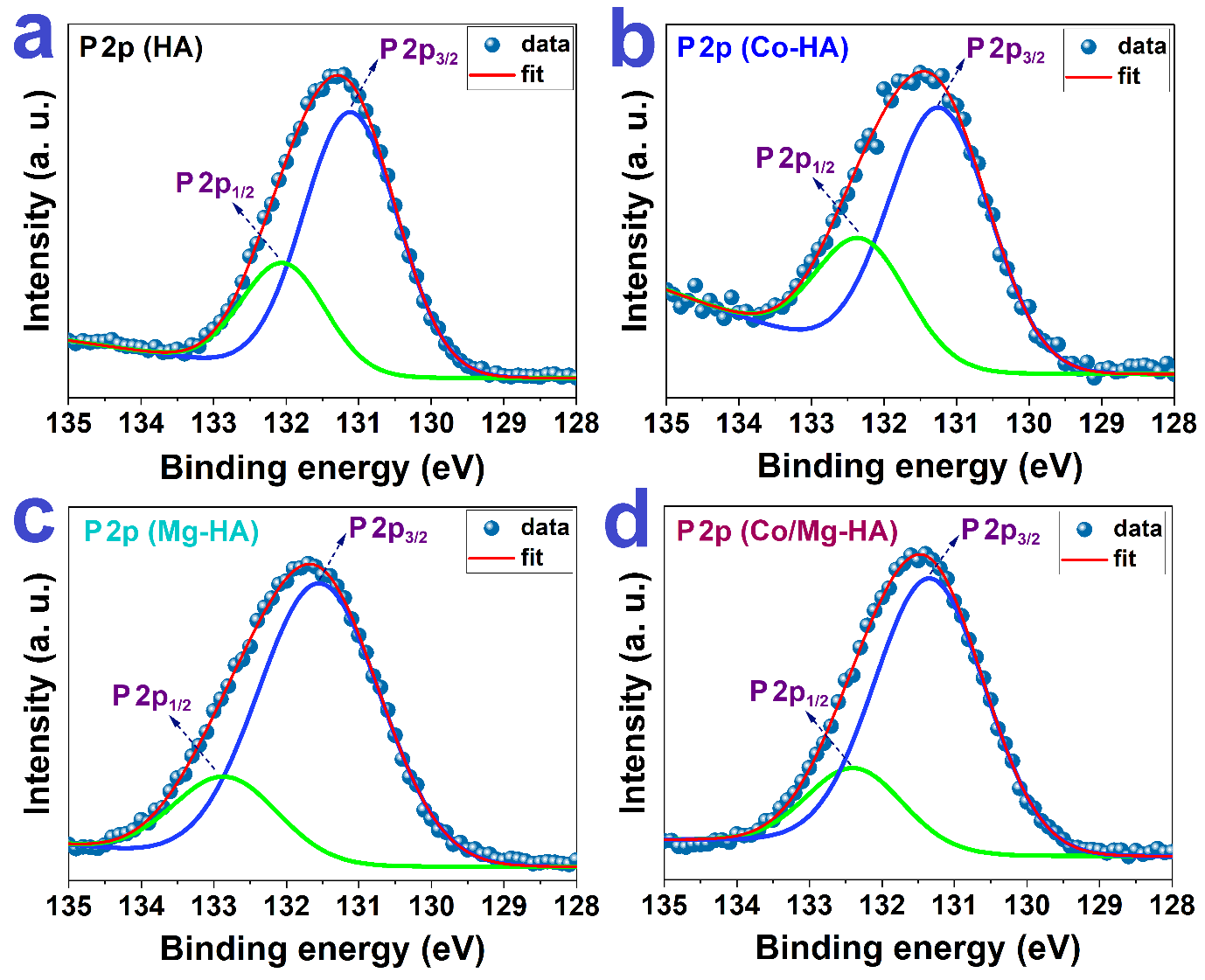


Figure S8. High-resolution XPS spectra of the P 2p region of (a) HA, (b) Co-HA, (c) Mg-HA, and (d) Co/Mg-HA nanoparticles.


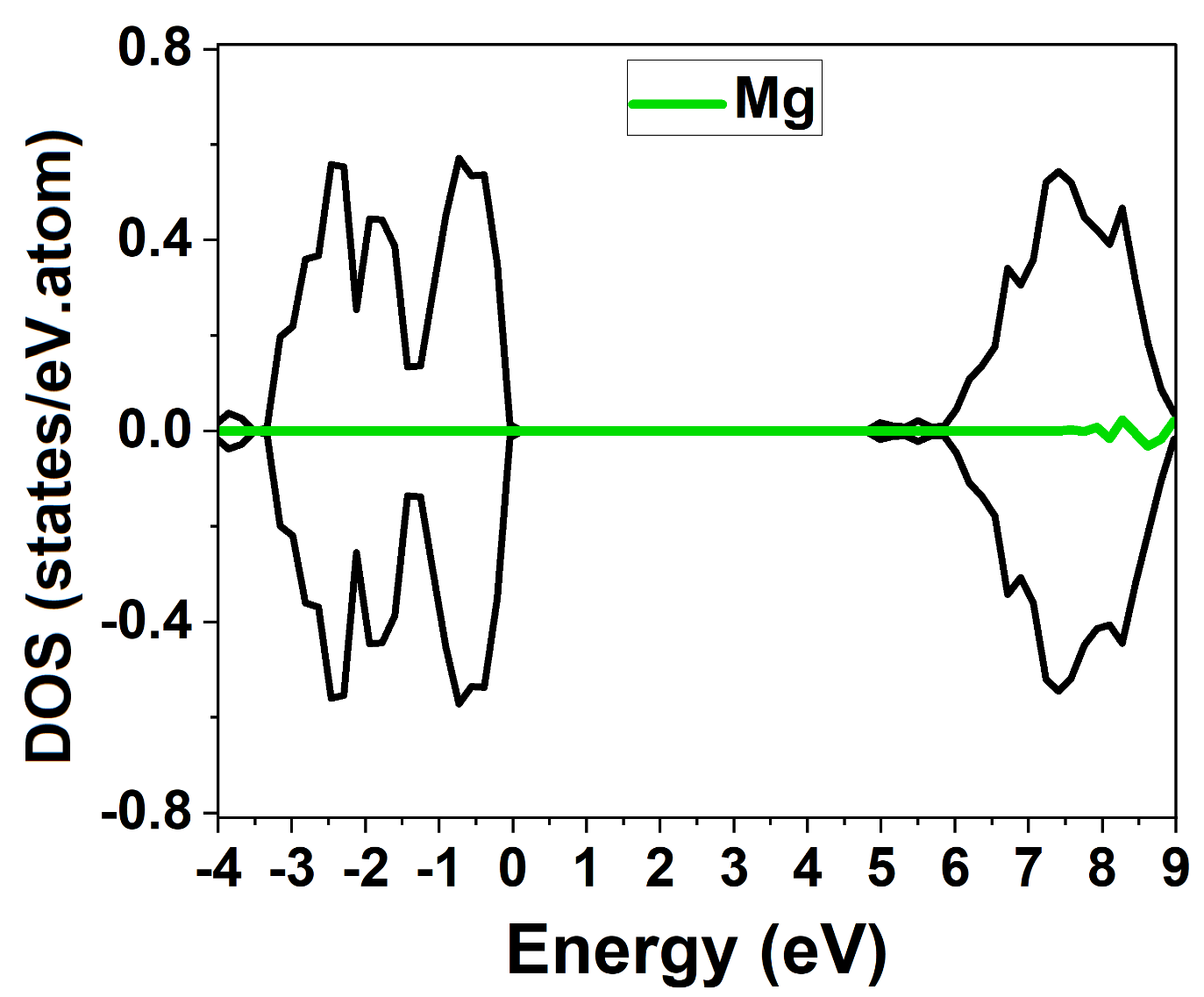


Figure S9. Density of states (DOS) diagram of Mg-doped HA cell structure.

Table S1. Relevant parameters from the Ca L_3_-edge spectra

| Sample | Peak a_1_ position (eV) | Peak a_1_  FWHM | Peak a_2_ position (eV) | Peak a_2_  FWHM | ΔL_3_ (a_2_-a_1_) (eV) | Relative peak intensity (a_1_/a_2_) |
| --- | --- | --- | --- | --- | --- | --- |
| HA | 348.106 | 0.343 | 349.1665 | 0.422 | 1.060 | 0.225 |
| Co-HA | 348.065 | 0.312 | 349.1636 | 0.414 | 1.098 | 0.304 |
| Mg-HA | 348.024 | 0.311 | 349.1665 | 0.391 | 1.142 | 0.332 |


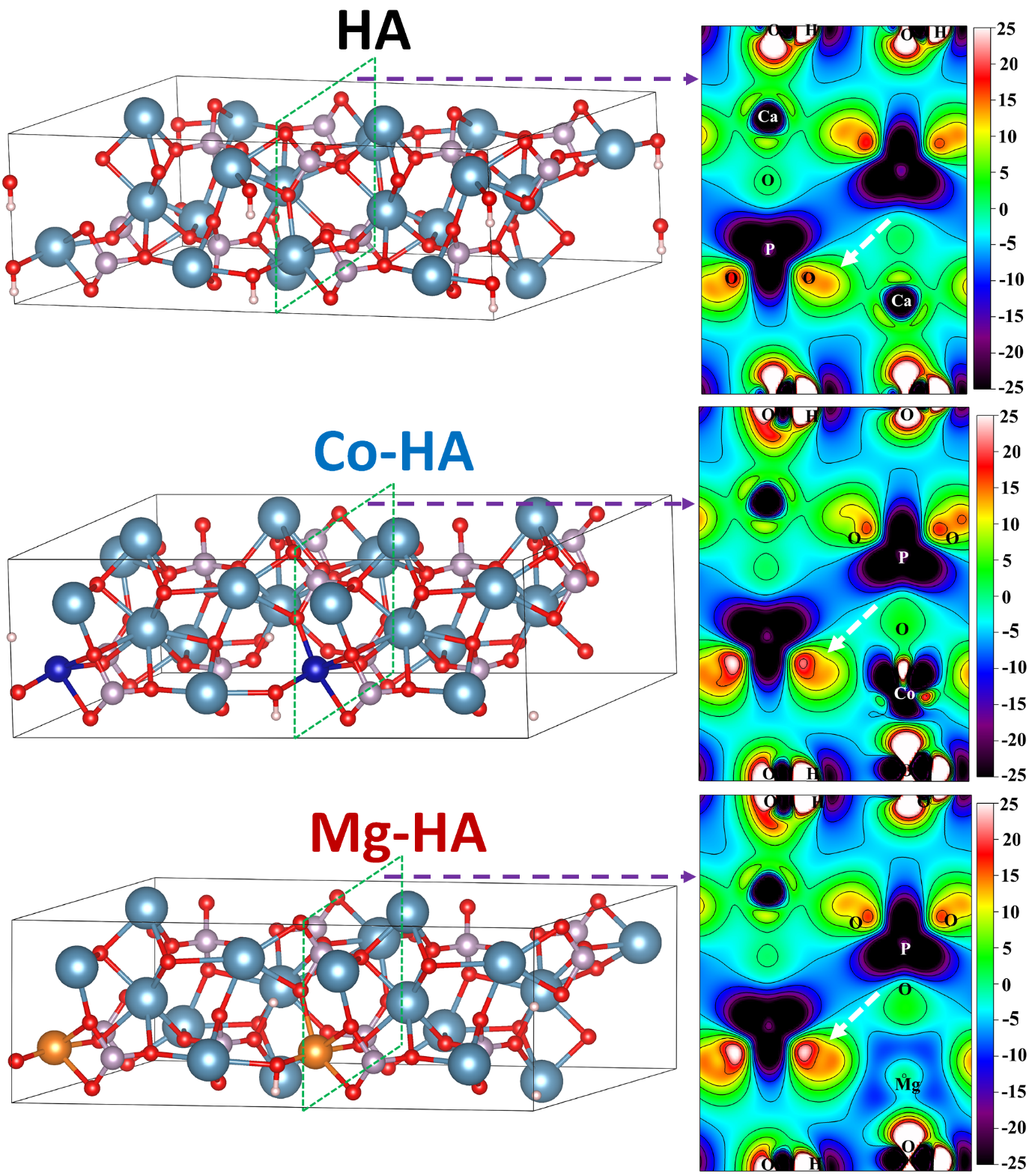


Figure S10. Two-dimensional charge density difference (2D CDD) for (001) plane of HA, Co-doped HA, and Mg-doped HA crystal cells.


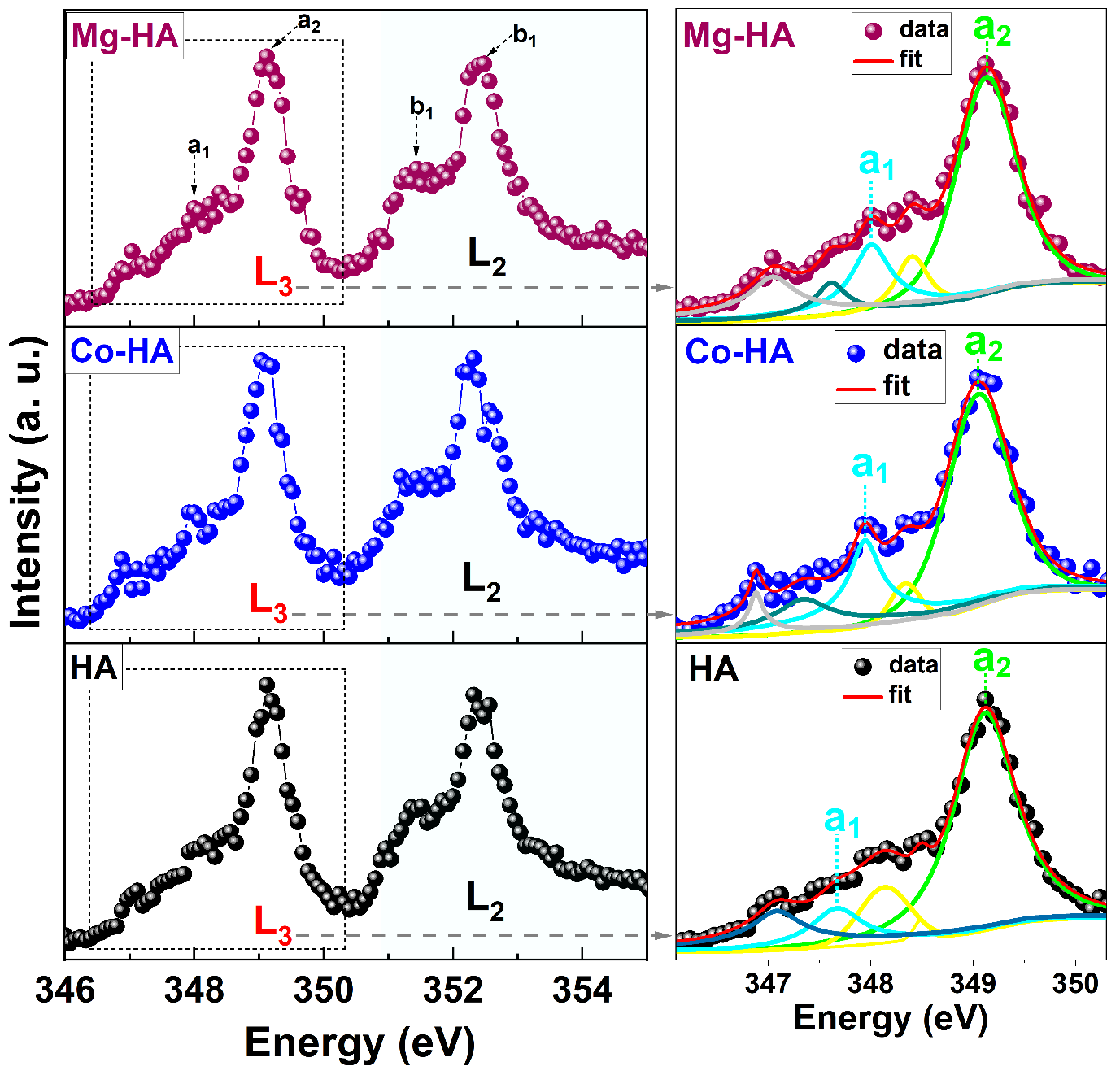


Figure S11. Calcium L-edge TEY XANES spectra of HA, Co-HA, and Mg-HA nanoparticles.

Table S2. Relevant parameters from the Ca L_3_-edge spectra (TEY)

| Sample | Peak a_1_ position (eV) | Peak a_1_  FWHM | Peak a_2_ position (eV) | Peak a_2_  FWHM | ΔL_3_ (a_2_-a_1_) (eV) | Relative peak intensity (a_1_/a_2_) |
| --- | --- | --- | --- | --- | --- | --- |
| HA | 348.137 | 0.580 | 349.117 | 0.722 | 0.979 | 0.255 |
| Co-HA | 347.945 | 0.424 | 349.038 | 0.709 | 1.093 | 0.408 |
| Mg-HA | 348.005 | 0.473 | 349.128 | 0.697 | 1.122 | 0.311 |


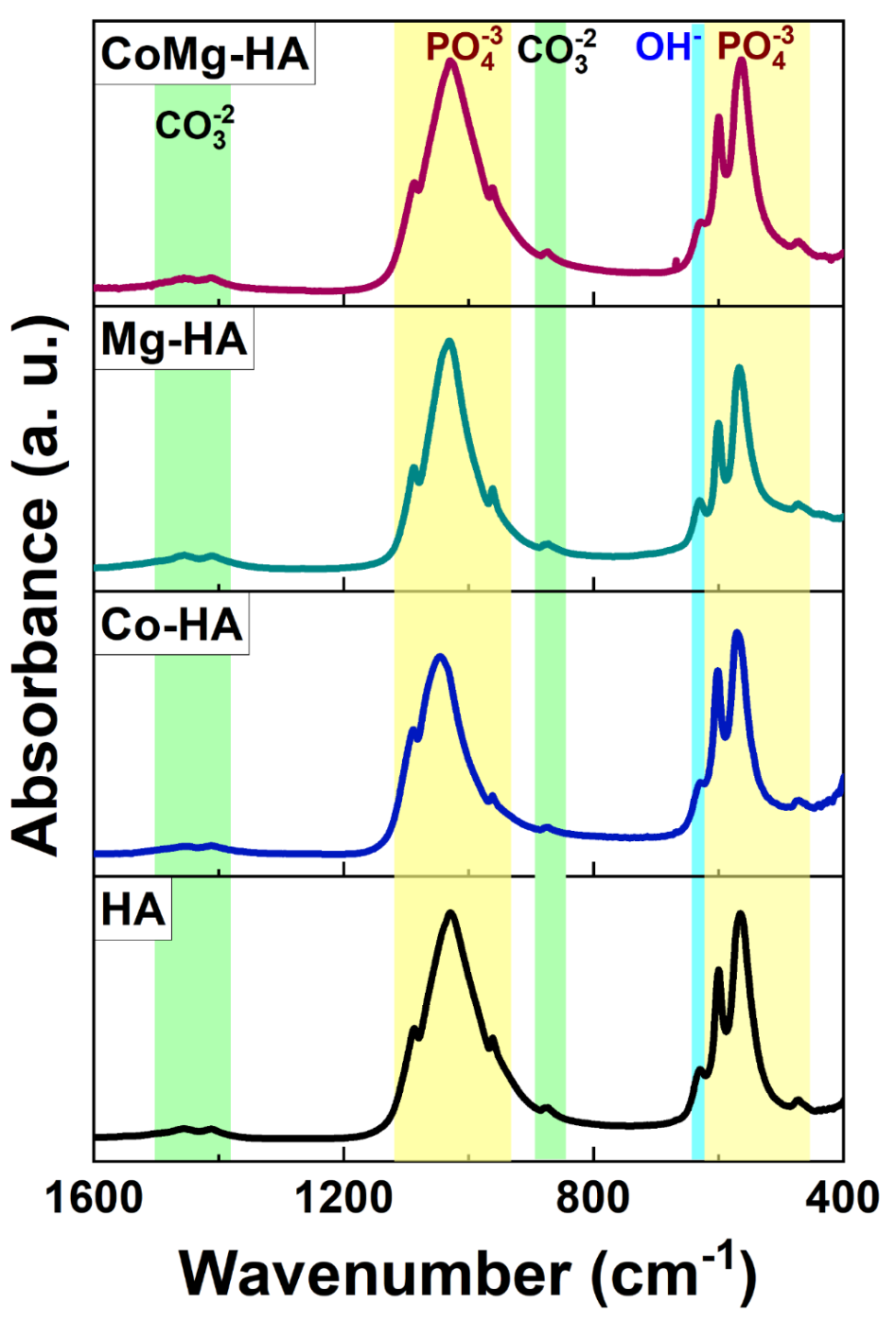


Figure S12. FTIR spectra of HA, Co-HA, Mg-HA, and Co/Mg-HA nanoparticles.


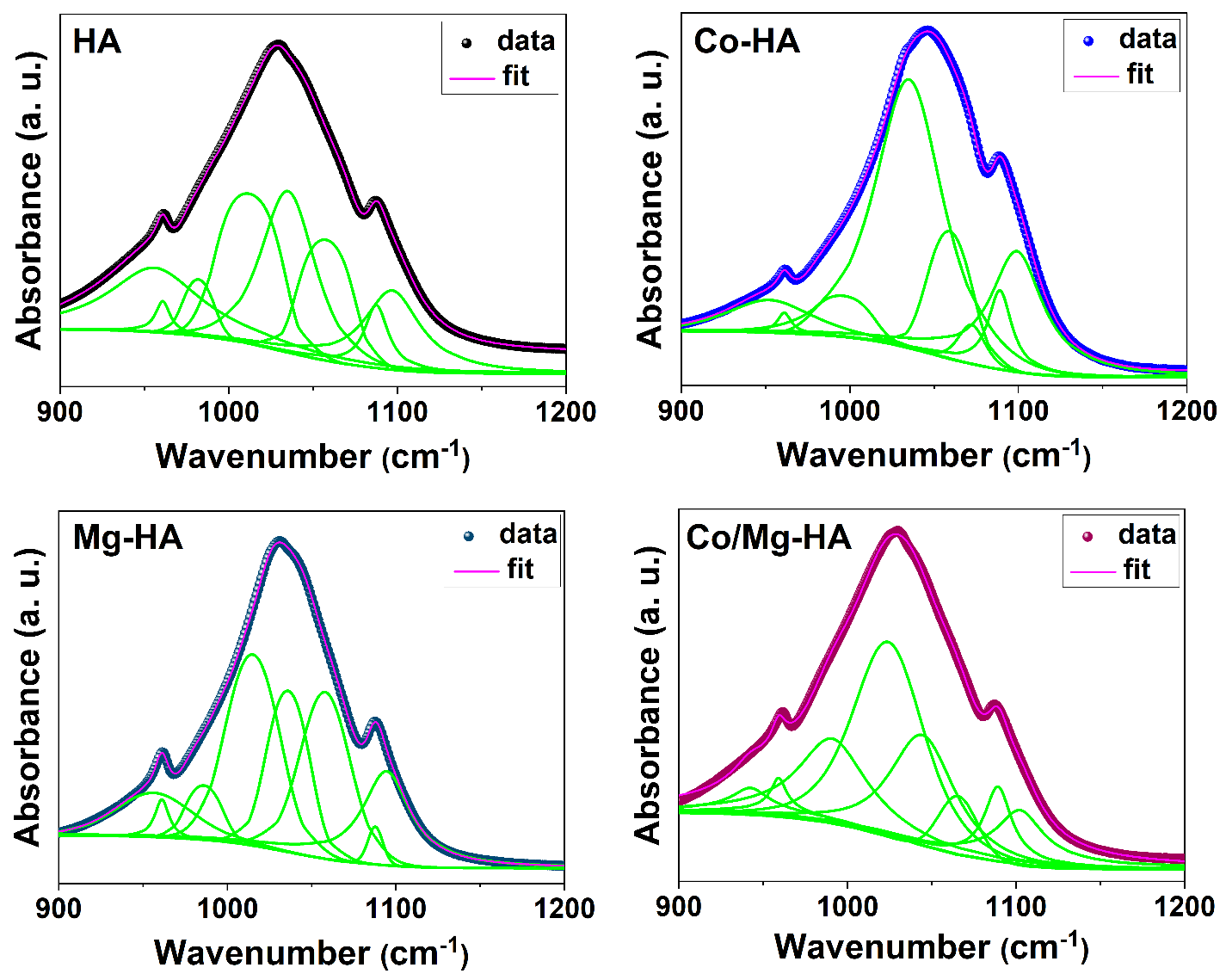


Figure S13. Deconvoluted FTIR in the 900-1200 cm^-1^ spectral region.


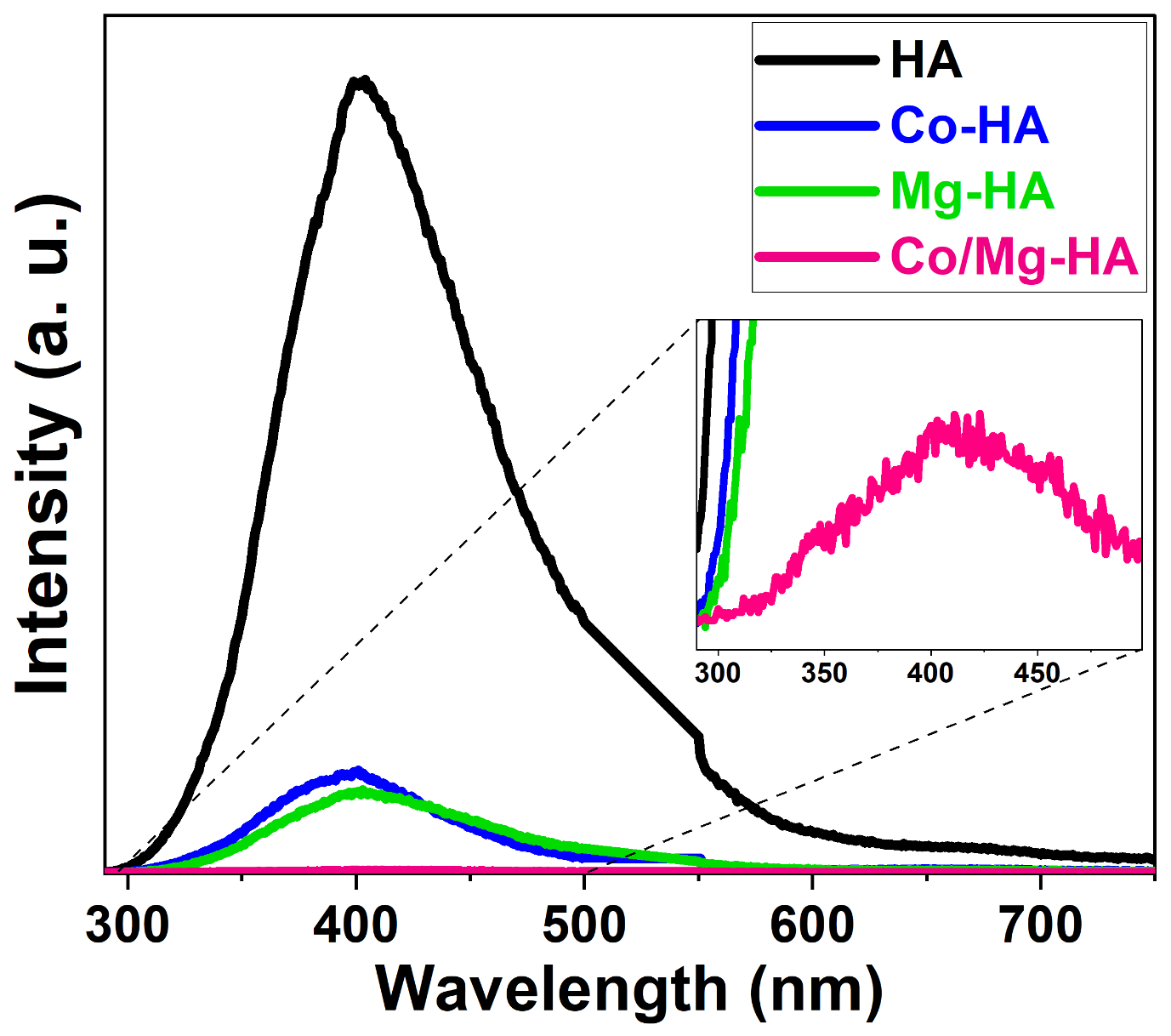


Figure S14. PL emission spectra of HA, Co-HA, Mg-HA, and Co/Mg-HA nanoparticles.


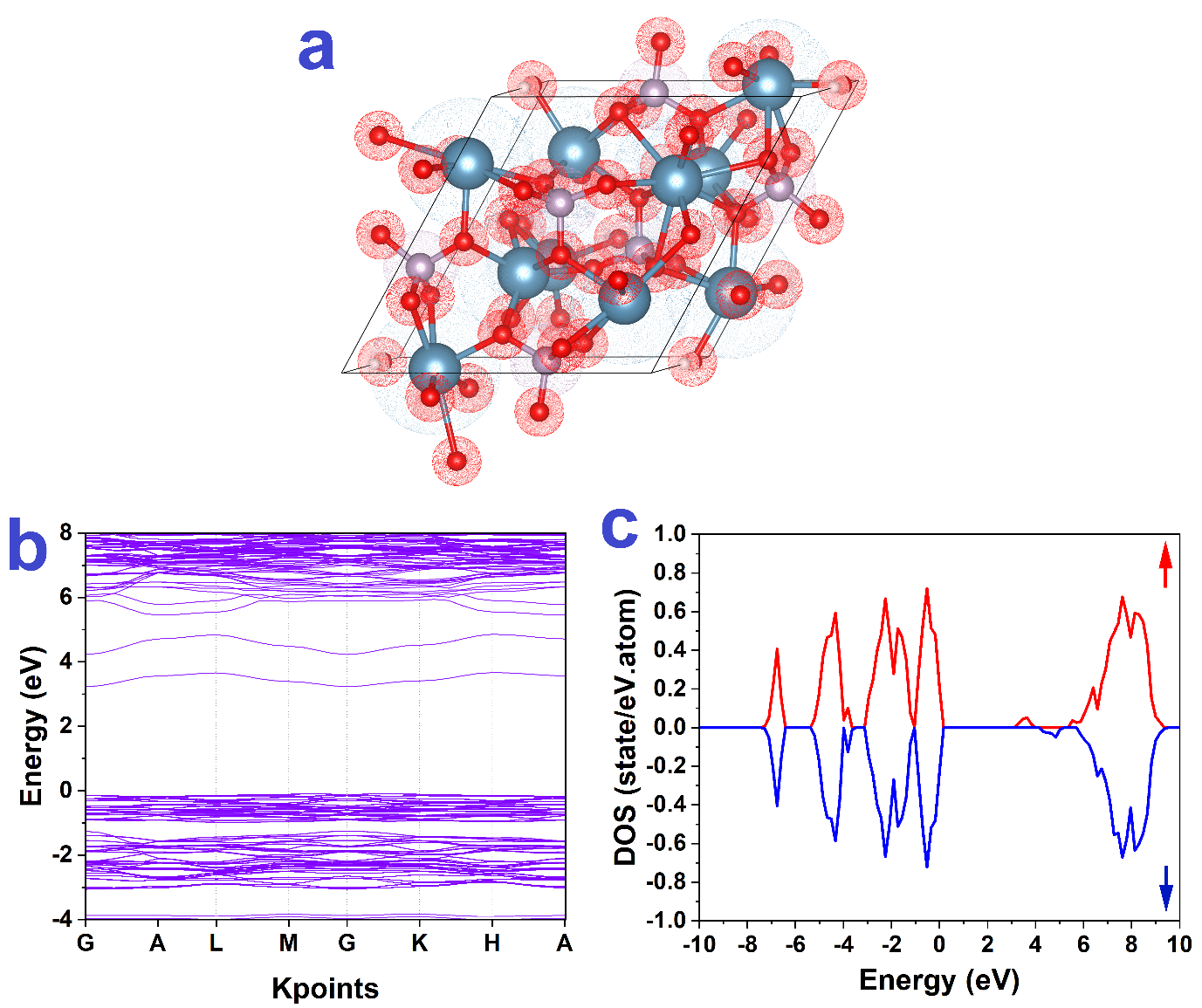


Figure S15. (a) 3D model of HA with O vacancy (HA-OV), (b) electronic band structure, and (c) a density states diagram of HA-OV crystal cell.


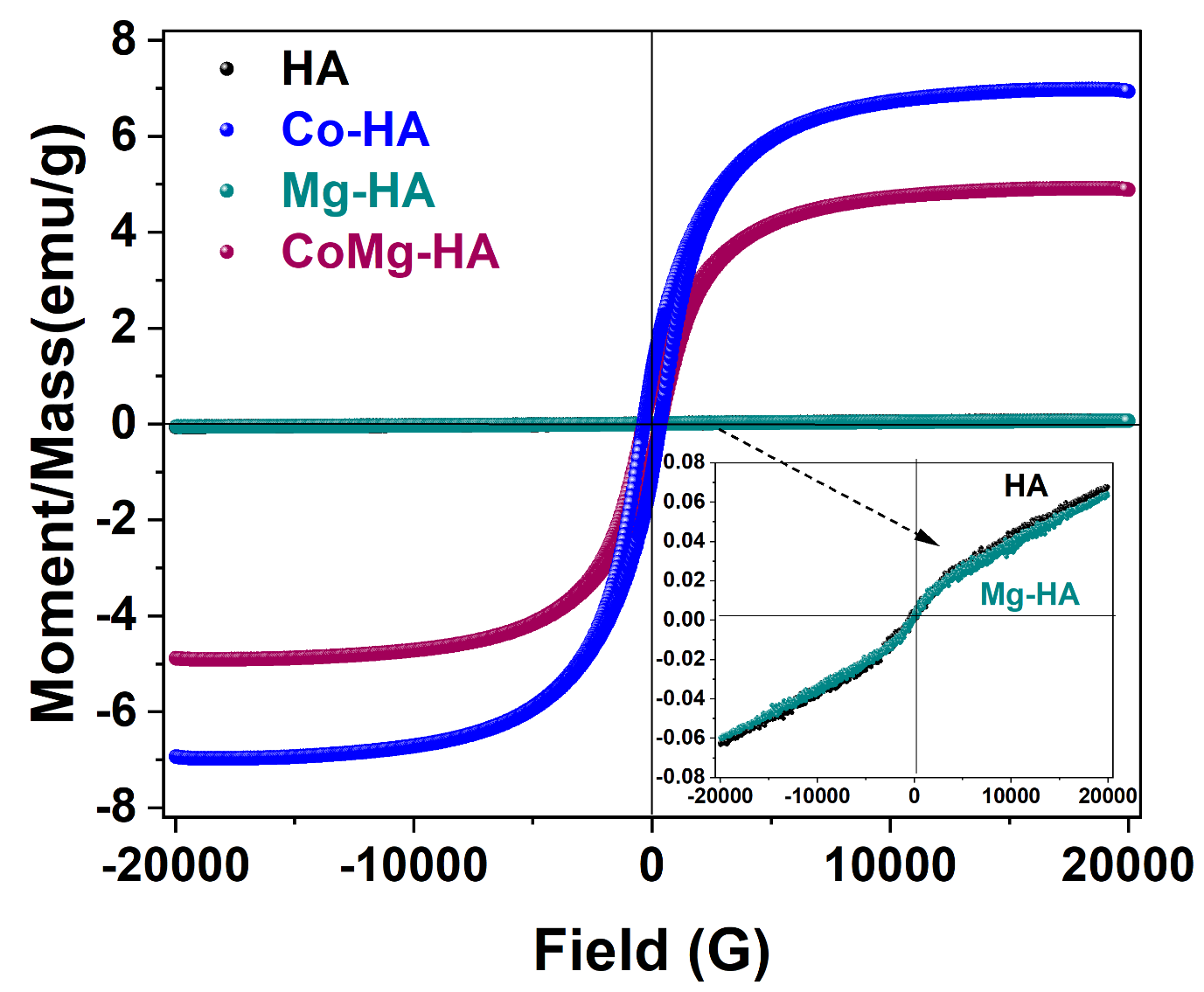


Figure S16. Magnetic hysteresis loops of the four nanoparticles.


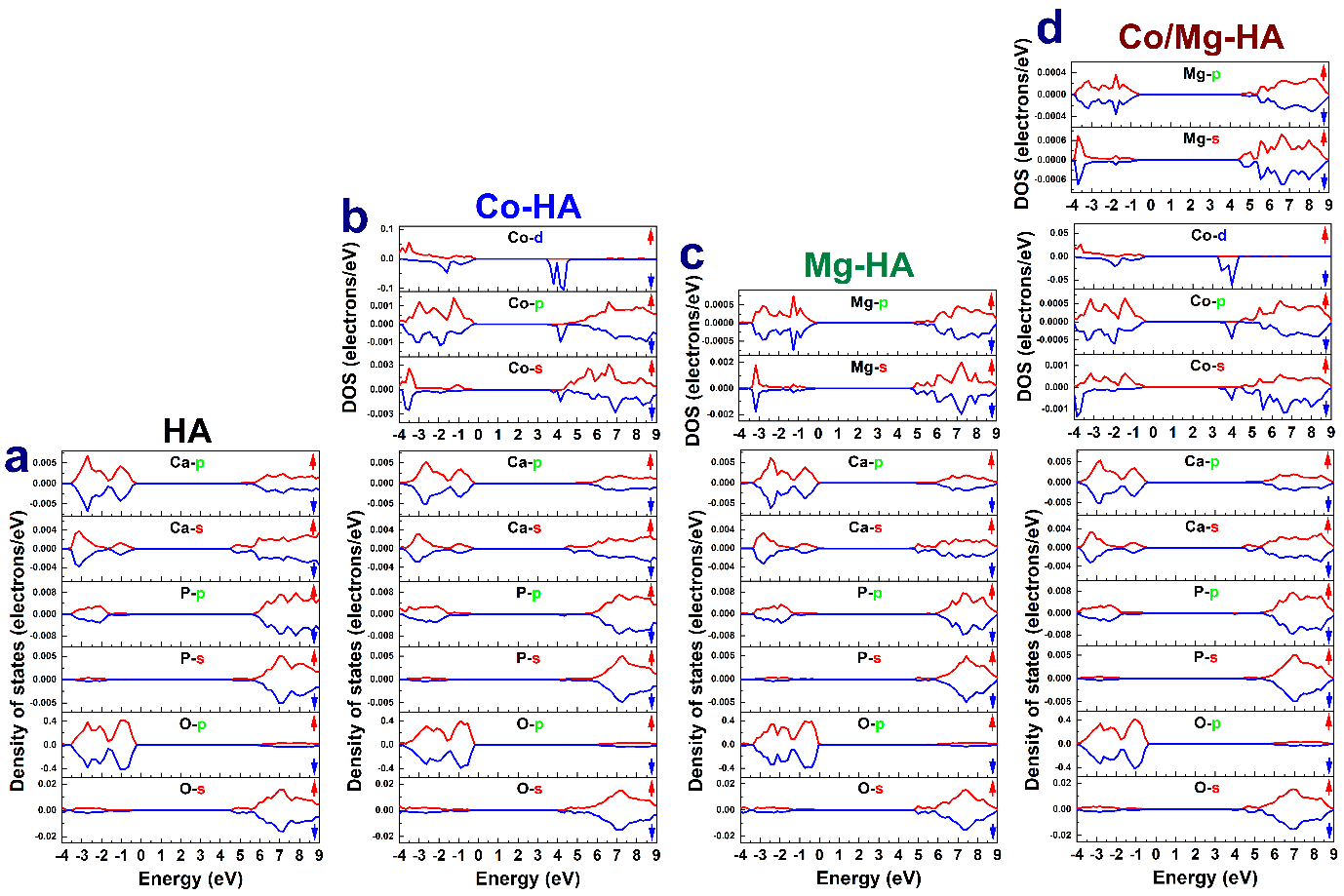


Figure S17. Projected density of states (PDOS) of (a) HA, (b) Co-HA, (c) Mg-HA, and (d) Co/Mg-HA systems.


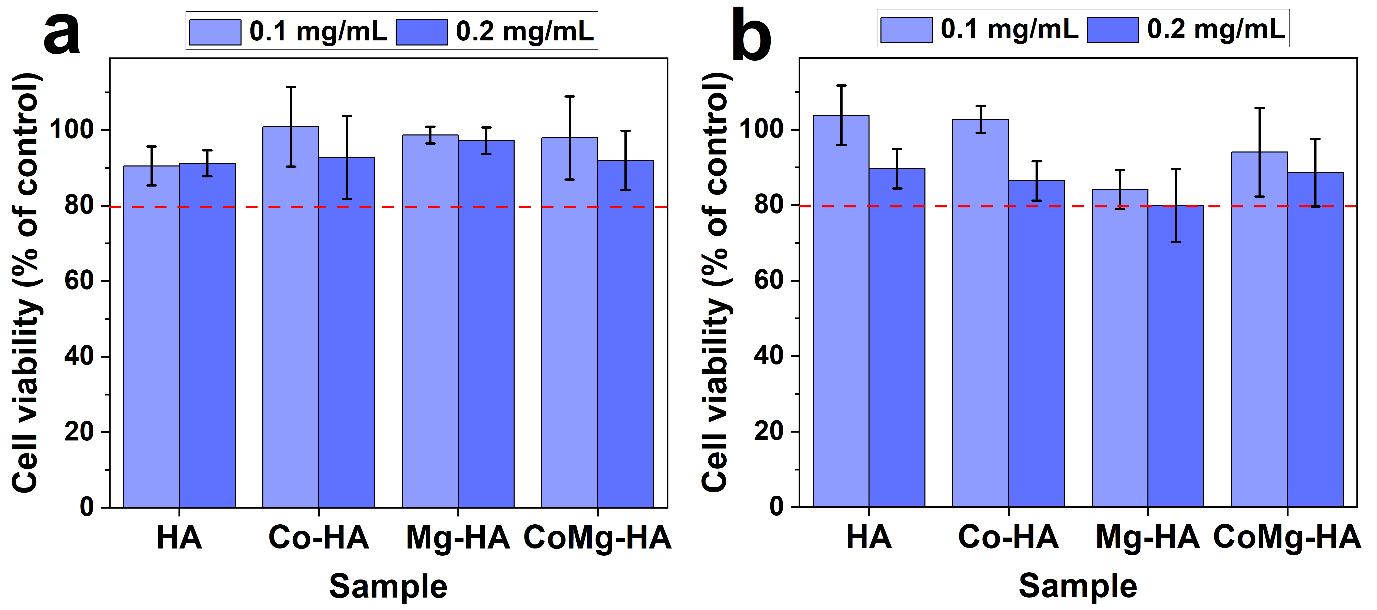


Figure S18. Cell viability using the AlamarBlue assay for culture treated by HA, Co-HA, Mg-HA, and Co/Mg-HA nanoparticles at two concentrations of 0.1 and 0.2 mg/mL at a) 12 and b) 24 h. (The data are presented as the mean ± SD.)


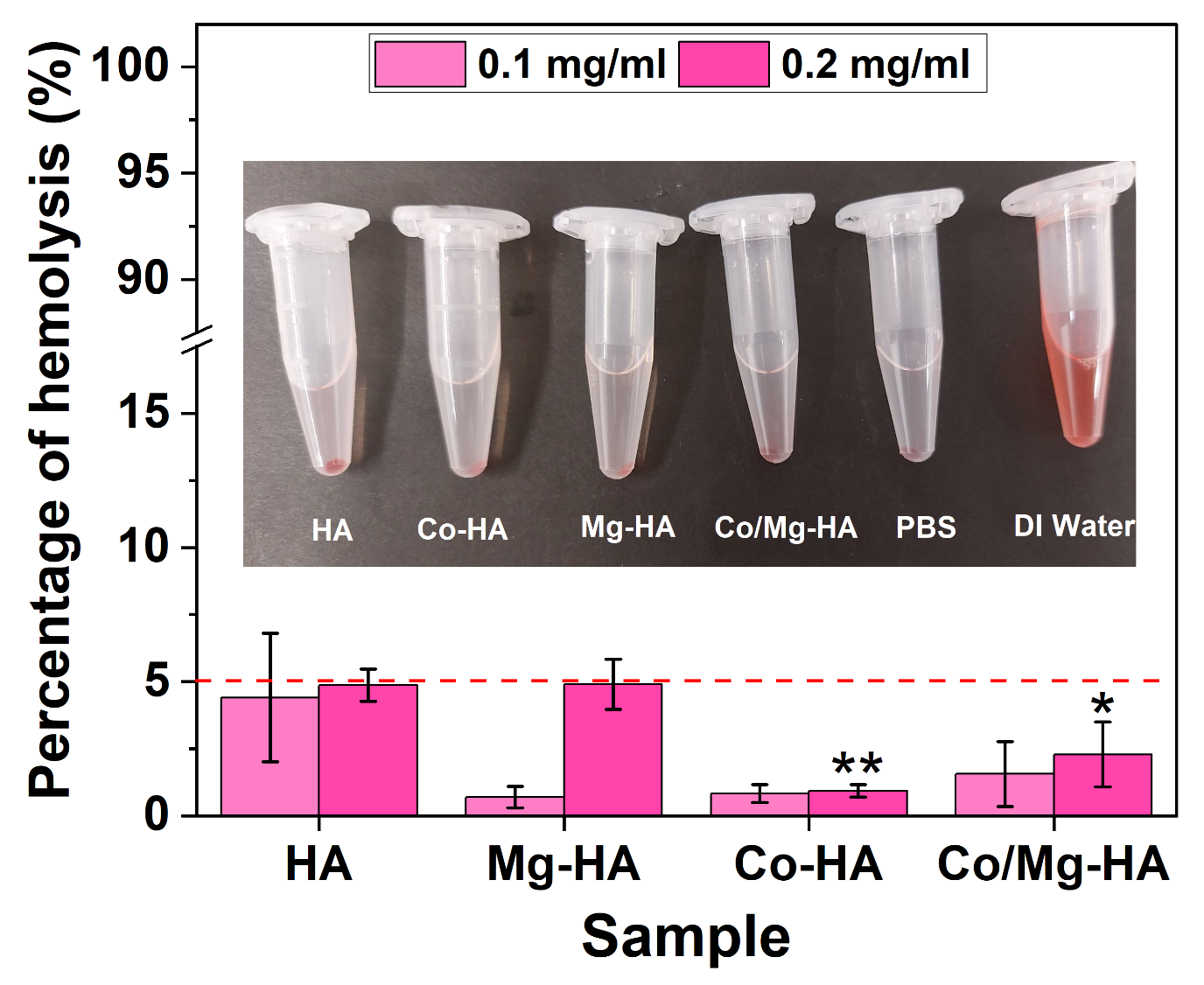


Figure S19. Hemolysis percentages of nanoparticles at concentrations of 0.1 and 0.2 mg/mL. (The data are presented as the mean ± SD. *p < 0.05 and **p < 0.01.)

1. * Corresponding author (H. Maleki-Ghaleh). E-mail address: [hmghaleh@ichf.edu.pl](mailto:hmghaleh@ichf.edu.pl) [↑](#footnote-ref-1)
2. ** Corresponding author (J. Paczesny). E-mail address: [jpaczesny@ichf.edu.pl](mailto:jpaczesny@ichf.edu.pl) [↑](#footnote-ref-2)
